# Supplementary figures and images for: Bile acid synthesis, modulation, and dementia: A metabolomic, transcriptomic, and pharmacoepidemiologic study
Source: PLoS Med. 2021 May 27;18(5):e1003615. doi: 10.1371/journal.pmed.1003615 (PMC8158920; doi:10.1371/journal.pmed.1003615)

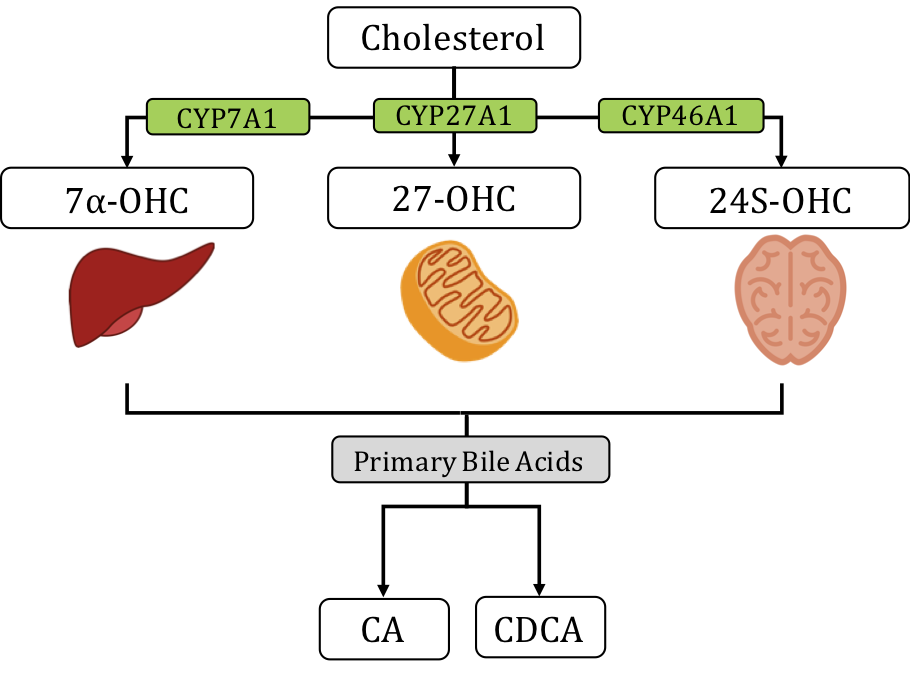

Supplement: S1 Fig — The oxidative catabolism of cholesterol occurs through 3 enzymatically catalyzed biochemical pathways: the classic/neutral pathway in the liver accounts for the majority of BA synthesis in humans and begins with the oxidation of cholesterol to 7α-OHC by microsomal CYP7A1, the rate-limiting enzyme of the pathway. The alternative or acidic pathway is responsible for synthesis of a smaller proportion of the BA pool; cholesterol is oxidized to 27-OHC, catalyzed by mitochondrial CYP27A1 in both liver and extra-hepatic tissues. Both the classic/neutral and acidic pathways of BA synthesis ultimately generate the primary BAs, CA, and CDCA which are the principal catabolic products of cholesterol. A third, neuron-specific pathway of cholesterol breakdown in the brain is catalyzed by CYP46A1-mediated conversion of cholesterol to 24S-OHC which effluxes into the peripheral circulation for further conversion into the primary BAs in the liver [31,40,57]. 7α-OHC, 7α-hydroxycholesterol; 24S-OHC, 24S-hydroxycholesterol; 27-OHC, 27-hydroxycholesterol; BA, bile acid; CA, cholic acid; CDCA, chenodeoxycholic acid. (TIFF) [file pmed.1003615.s001.tiff]
