# Supplementary material for: Bile acid synthesis, modulation, and dementia: A metabolomic, transcriptomic, and pharmacoepidemiologic study
Source: PLoS Med. 2021 May 27;18(5):e1003615. doi: 10.1371/journal.pmed.1003615 (PMC8158920; doi:10.1371/journal.pmed.1003615)
Supplement: S1 Text — (DOCX) [file pmed.1003615.s002.docx]

**Supplementary Text**

*BLSA-NI visit schedule*

BLSA-NI imaging and visit schedules have varied over time [1,2]; participant visits in this study occurred after 2009 when participants between the ages of 60-79 had biennial imaging assessments in addition to clinical, neurological, and laboratory evaluations. Participants 80 years and older received annual assessments [1].

*BLSA Quantitative serum metabolomics assays*

Quantitative metabolomics assays were performed on serum samples collected after 2009 among BLSA-NI participants after an overnight fast to measure concentration of 7α-OHC using the Biocrates kit filter plate and to measure concentrations of cholic acid (CA) and chenodeoxycholic acid (CDCA) using the Biocrates Bile Acids kit. Details on both assay kits, as well as calibration steps have been published previously [3].

*Biocrates internal standards*

A defined, consistent amount of internal standard was applied to each sample before and calibration standard early in the sample preparation. An external 7-point calibration was included in each measurement unit. After measurement, peaks were extracted using Waters MassLynx software or Sciex Analyst software. The peak annotation for the samples depended on the relation of retention time of the analyte peak signal to the peak signal of the internal standard. The target relation between these two factors for the samples was specified by the relation of both factors observed in the calibrator. The mass tolerance of the quadrupole devices was around 0.1 m/z. The retention time tolerance was below 0.02 min. The calibration curve is constructed as a plot of X = ratio of the concentration of analyte to concentration of internal standard versus Y = ratio of area of analyte to the internal standard. For application, the ratio of analyte to internal standard area is determined for the samples and the equation for the regression line then allows determination of sample concentration. If the sample volume used deviated from the target volume of the assay, a factor was included in the calculation to take the volume difference into account.

*Calculating and quantifying white matter lesions (WMLs) and brain volumes (BLSA & ADNI)*

WMLs as well as global and regional brain volumes were calculated using Multi-atlas Region Segmentation Utilizing Ensemble (MUSE) [4]. A detailed description of MRI acquisition and preprocessing procedures has been published previously [2]. To calculate and quantify white matter lesions (WMLs), a supervised learning-based multi-modal lesion segmentation technique was applied; methods have been described in detail previously [5]. Briefly, the method involved co-registration of T1-weighted, T2-weighted, Proton Density and FLAIR scans, histogram normalization to a template image, feature extraction, voxel-wise label assignment using a model trained on manually segmented lesions, and false-positive elimination. WML burden is calculated by summing lesion volumes in frontal, temporal, parietal and occipital white matter regions of interest.

*Statistical methods: Step 1: Test associations between cholesterol catabolism (i.e. bile acid synthesis) and neuroimaging markers of dementia (BLSA & ADNI)*

Time was defined as years between baseline and follow-up visits. In BLSA, the fixed effects in the model included the following predictors: metabolite concentration, mean-centered baseline age, race, and baseline intracranial volume, time and the two-way interactions of mean-centered baseline age with time and metabolite concentration with time. In ADNI we used the same fixed effects and included the testing site and whether individuals were from ADNI1, ADNI2, or ADNIGO as additional covariates. In BLSA, due to restricted sample size and follow-up visits, we only included a random effect for intercept. In ADNI we included random effects for both intercept and slope and modeled the covariance structure as unstructured. For WMLs, we reported significant associations at the p < 0.05 level. For brain volumes, we conservatively reported significant associations at the FDR-corrected p < 0.05 level [6,7] by correcting p-values across all brain regions and metabolites (18 (brain regions) x 3 (metabolites) = 54 comparisons for BLSA and 12 (brain regions) x 2 (metabolites) = 24 comparisons for ADNI). In order to easily compare results across all steps we reported all associations with “lower” metabolite concentration. For WMLs, a significant negative coefficient for the predictor of interest (metabolite concentration x time) indicates that a lower metabolite concentration is associated with faster rates of WML accumulation; a positive coefficient indicates lower metabolite concentration is associated with slower rate of WML accumulation. For brain volumes, coefficient interpretations were reversed; significant positive and negative coefficients indicate that lower metabolite concentrations are associated with faster and slower rates brain atrophy, respectively. Coefficient interpretations for ventricular volume change were similar to WML accumulation. To simplify, in the Results tables in the main text coefficients in green indicate that lower serum concentration of the metabolite is significantly associated with higher levels of brain amyloid-β, faster accumulation of WML accumulation, or faster brain atrophy. Coefficients in red indicate that lower serum concentration of the metabolite is significantly associated with lower levels of brain amyloid-β, slower accumulation of WML, or slower brain atrophy. Statistical analyses were performed using STATA version 13.0.

*Statistical methods: Step 2: Test whether pharmacological modulation of bile acids alters dementia risk in a large, real-world clinical dataset*

We tested the effect of cumulative BAS use, using time-varying variables based on the number of prescriptions (2, 3-5, and ≥6) as compared to ≥2 prescriptions of LMT. Age was the time scale of the analyses; follow-up time started at the patient age at one year after the 2^nd^ BAS or LMT prescription and ended at age of first dementia record of any type, treatment crossover, death, transfer out of clinic, practice last collection date, or end of study (August 1^st^, 2018).

Models were adjusted for factors that were significantly different between BAS and LMT groups to account for potential confounding by indication. These factors included smoking status, body mass index (BMI), alcohol consumption, and prior metformin use, coronary artery diseases, type 2 diabetes, dyslipidemia, and cancer history. We additionally adjusted for statin use during follow-up (until one year before exit date) as a time-varying variable. The baseline hazard in the Cox models was stratified by matched set to account for matched design. We further evaluated whether the risk for dementia differs by sex by comparing the regression coefficient of BAS predicating VaD for males and females using the TEST statement in PROC PHREG procedure in SAS. To examine the possible dose-response relationships (i.e. higher BAS use was associated with increased risk of dementia), tests for linear trend were employed. Missing values for smoking, alcohol and BMI were imputed using IVEware, Version 0.3 with 10 iteration cycles to produce 5 imputed datasets [8]. Results were combined across multiple imputed datasets using PROC Mianalyse. All statistical analyses were conducted using SAS, version 9.4 (SAS Institute, Cary, NC) and statistical significance were defined as two-sided p<0.05.

Analytic code is available upon request. The study protocol was approved by the CPRD Independent Scientific Advisory Committee (Protocol #18_173). The use of CPRD database was exempted from IRB review by the Office for Human Research Protections at the National Institutes of Health because of the anonymized nature of the database.

*Study participants: BLSA autopsy study*

Participants indicated as AD had clinical symptoms (i.e., cognitive impairment) of AD during life assessed by longitudinal cognitive assessments at consensus diagnosis conferences [9] and had AD pathology at autopsy, determined by an expert neneuropathologist. Participants indicated as control (CON) had no clinical symptoms during life and no AD pathology at autopsy. Tissue samples from AD (n = 16) and CON (n = 13) samples were extracted from the cortical surface of the autopsied brains using a 4-mm diameter tissue punch from the inferior temporal gyrus (ITG) and middle frontal gyrus (MFG), regions representing areas of early neurofibrillary (i.e. tau) and neuritic plaque (i.e. amyloid) accumulation respectively [10,11], as well as the cerebellum (CB). All tissue samples were stored at -80C until metabolomic assays.

**References**

1. Armstrong NM, An Y, Beason-Held L, Doshi J, Erus G, Ferrucci L, et al. Sex differences in brain aging and predictors of neurodegeneration in cognitively healthy older adults. Neurobiology of aging. 2019;81:146-56. Epub 2019/07/08. doi: 10.1016/j.neurobiolaging.2019.05.020. PubMed PMID: 31280118.

2. Resnick SM, Pham DL, Kraut MA, Zonderman AB, Davatzikos C. Longitudinal magnetic resonance imaging studies of older adults: a shrinking brain. J Neurosci. 2003;23(8):3295-301. Epub 2003/04/30. PubMed PMID: 12716936; PubMed Central PMCID: PMCPMC6742337.

3. Mahajan UV, Varma VR, Huang CW, An Y, Tanaka T, Ferrucci L, et al. Blood Metabolite Signatures of Metabolic Syndrome in Two Cross-Cultural Older Adult Cohorts. International journal of molecular sciences. 2020;21(4). Epub 2020/02/23. doi: 10.3390/ijms21041324. PubMed PMID: 32079087; PubMed Central PMCID: PMCPMC7072935.

4. Doshi J, Erus G, Ou Y, Resnick SM, Gur RC, Gur RE, et al. MUSE: MUlti-atlas region Segmentation utilizing Ensembles of registration algorithms and parameters, and locally optimal atlas selection. Neuroimage. 2016;127:186-95. Epub 2015/12/19. doi: 10.1016/j.neuroimage.2015.11.073. PubMed PMID: 26679328; PubMed Central PMCID: PMCPMC4806537.

5. Zacharaki EI, Kanterakis S, Bryan RN, Davatzikos C. Measuring brain lesion progression with a supervised tissue classification system. Med Image Comput Comput Assist Interv. 2008;11(Pt 1):620-7. Epub 2008/11/05. doi: 10.1007/978-3-540-85988-8_74. PubMed PMID: 18979798.

6. W. H. Benjamini–Hochberg Method. In: W. D, O. W, KH. C, H. Y, editors. Encyclopedia of Systems Biology. New York, NY: Springer; 2013.

7. Benjamini Y, Hochberg Y. Controlling the False Discovery Rate: A Practical and Powerful Approach to Multiple Testing. Journal of the Royal Statistical Society Series B (Methodological). 1995;57(1):289-300.

8. Raghunathan TE, Solenberger PW, Van Hoewyk J. IVEware: Imputation and Variance Estimation Software User Guide. 2002.

9. Kawas C, Gray S, Brookmeyer R, Fozard J, Zonderman A. Age-specific incidence rates of Alzheimer's disease: the Baltimore Longitudinal Study of Aging. Neurology. 2000;54(11):2072-7. PubMed PMID: 10851365.

10. Buckley RF, Hanseeuw B, Schultz AP, Vannini P, Aghjayan SL, Properzi MJ, et al. Region-Specific Association of Subjective Cognitive Decline With Tauopathy Independent of Global beta-Amyloid Burden. JAMA Neurol. 2017;74(12):1455-63. Epub 2017/10/04. doi: 10.1001/jamaneurol.2017.2216. PubMed PMID: 28973551; PubMed Central PMCID: PMCPMC5774633.

11. Li Y, Rinne JO, Mosconi L, Pirraglia E, Rusinek H, DeSanti S, et al. Regional analysis of FDG and PIB-PET images in normal aging, mild cognitive impairment, and Alzheimer's disease. European journal of nuclear medicine and molecular imaging. 2008;35(12):2169-81. Epub 2008/06/21. doi: 10.1007/s00259-008-0833-y. PubMed PMID: 18566819; PubMed Central PMCID: PMCPMC2693402.
