# Supplementary material for: Bile acid synthesis, modulation, and dementia: A metabolomic, transcriptomic, and pharmacoepidemiologic study
Source: PLoS Med. 2021 May 27;18(5):e1003615. doi: 10.1371/journal.pmed.1003615 (PMC8158920; doi:10.1371/journal.pmed.1003615)
Supplement: S2 Table — Indicates data availability in the scRNA-Seq ROSMAP dataset. BA receptor genes that are indicated as “Not Available” either did not have sufficient counts or did not have any data available in the ROSMAP scRNA-Seq dataset. BA, bile acid; CHRM2, Cholinergic Receptor Muscarinic 2; CHRM3, Cholinergic Receptor Muscarinic 3; FGF19, Fibroblast Growth Factor 19; FPR1, Formyl Peptide Receptor 1; GPBAR1, G Protein-Coupled Bile Acid Receptor 1; HNF4A, Hepatocyte Nuclear Factor 4 Alpha; KDR, Kinase Insert Domain Receptor; NR0B2, Nuclear Receptor Subfamily 0 Group B Member 2; NR1H2, Nuclear Receptor Subfamily 1 Group H Member 2; NR1H3, Nuclear Receptor Subfamily 1 Group H Member 3; NR1H4, Nuclear Receptor Subfamily 1 Group H Member 4; NR1I2, Nuclear Receptor Subfamily 1 Group I Member 2; NR1I3, Nuclear Receptor Subfamily 1 Group I Member 3; NR3C1, Nuclear Receptor Subfamily 3 Group C Member 1; NR5A2, Nuclear Receptor Subfamily 5 Group A Member 2; PPARA, Peroxisome Proliferator Activated Receptor Alpha; PPARD, Peroxisome Proliferator Activated Receptor Delta; PPARG, Peroxisome Proliferator Activated Receptor Gamma; RARA, Retinoic Acid Receptor Alpha; ROSMAP, Religious Orders Study and Memory and Aging Project; RXRA, Retinoid X Receptor Alpha; RXRB, Retinoid X Receptor Beta; RXRG, Retinoid X Receptor Gamma; S1PR2, Sphingosine-1-Phosphate Receptor 2; scRNA-Seq, single-cell RNA sequencing; VDR, Vitamin D Receptor. (DOCX) [file pmed.1003615.s004.docx]

**Supplementary Table 2. ROSMAP scRNA-Seq BA receptor gene expression data availability**

|  | **Available** | **Not Available** |
| --- | --- | --- |
| NR1I3 | X |  |
| RXRG | X |  |
| NR5A2 | X |  |
| CHRM3 | X |  |
| GPBAR1 | X |  |
| PPARG | X |  |
| NR1I2 | X |  |
| KDR | X |  |
| NR3C1 | X |  |
| RXRB | X |  |
| PPARD | X |  |
| CHRM2 | X |  |
| RXRA | X |  |
| NR1H3 | X |  |
| VDR | X |  |
| NR1H4 | X |  |
| RARA | X |  |
| HNF4A | X |  |
| NR1H2 | X |  |
| FPR1 | X |  |
| PPARA | X |  |
| S1PR2 |  | X |
| FGF19 |  | X |
| NR0B2 |  | X |

Indicates data availability from scRNA-Seq ROSMAP dataset. Bile acid (BA) receptor genes that are indicated as “Not Available” either did not have sufficient counts or did not have any data available in the ROSMAP scRNA-Seq dataset.

NR1I3: Nuclear Receptor Subfamily 1 Group I Member 3; RXRG: Retinoid X Receptor Gamma; NR5A2: Nuclear Receptor Subfamily 5 Group A Member 2; CHRM3: Cholinergic Receptor Muscarinic 3; GPBAR1: G Protein-Coupled Bile Acid Receptor 1; PPARG: Peroxisome Proliferator Activated Receptor Gamma; NR1I2: Nuclear Receptor Subfamily 1 Group I Member 2; KDR: Kinase Insert Domain Receptor; NR3C1: Nuclear Receptor Subfamily 3 Group C Member 1; RXRB: Retinoid X Receptor Beta; PPARD: Peroxisome Proliferator Activated Receptor Delta; CHRM2: Cholinergic Receptor Muscarinic 2; RXRA: Retinoid X Receptor Alpha; NR1H3: Nuclear Receptor Subfamily 1 Group H Member 3; VDR: Vitamin D Receptor; NR1H4: Nuclear Receptor Subfamily 1 Group H Member 4; RARA: Retinoic Acid Receptor Alpha; HNF4A: Hepatocyte Nuclear Factor 4 Alpha; NR1H2: Nuclear Receptor Subfamily 1 Group H Member 2; FPR1: Formyl Peptide Receptor 1; PPARA: Peroxisome Proliferator Activated Receptor Alpha; S1PR2: Sphingosine-1-Phosphate Receptor 2; FGF19: Fibroblast Growth Factor 19; NR0B2: Nuclear Receptor Subfamily 0 Group B Member 2

**Supplementary Table 3a. Demographic characteristics of BLSA-NI sample**

|  | PiB Analyses | Brain atrophy/ WML analyses |
| --- | --- | --- |
| Sample size | 141 | 134 |
| Age, mean (SD) | 76.1 (8.5) | 76.1 (8.5) |
| Male Sex, n (% male) | 66 (46.8) | 62 (46.3) |
| White Race, n (% white) | 107 (75.9) | 102 (76.1) |
| Amyloid +ve, n (% +ve) | 36 (25.5) | - |
| APOE4, n (% e4) | 36 (27.1) | 35 (26.3) |
| Longitudinal MRI visits, n | - | 332 |
| Follow-up MRI visits, mean (SD) | - | 2.5 (1.5) |

BLSA: Baltimore Longitudinal Study of Aging; NI: Neuroimaging; WML: White Matter Lesions; PiB: Pittsburgh compound B; MRI: Magnetic Resonance Imaging; APOE4: e4 allele of the Apolipoprotein E gene; SD: standard deviation

**Supplementary Table 3b. Demographic characteristics of ADNI sample**

|  | Brain atrophy/ WML analyses |
| --- | --- |
| Sample size | 1666 |
| Age, mean (SD) | 73.8 (7.2) |
| Male Sex, n (% male) | 918 (55.1) |
| White Race, n (% white) | 1538 (92.5) |
| Longitudinal MRI visits, n | 8686 |
| Follow-up MRI visits, mean (SD) | 5.2 (2.5) |

ADNI: Alzheimer’s Disease Neuroimaging Initiative; NI: Neuroimaging; WML: White Matter Lesions; MRI: Magnetic Resonance Imaging; SD: standard deviation

**Supplementary Table 4. Associations between serum metabolite concentrations and PiB/ amyloid status**

|  | Total | | Male | | Female | |
| --- | --- | --- | --- | --- | --- | --- |
|  | coef | pval | coef | pval | coef | pval |
| 7α-hydroxycholesterol | -0.205 | 0.127 | -0.21 | 0.246 | -0.127 | 0.536 |
| Chenodeoxycholic acid | 0.002 | 0.996 | 0.236 | 0.598 | -0.186 | 0.669 |
| Cholic acid | 0.35 | 0.262 | 0.364 | 0.45 | 0.388 | 0.372 |

PiB: Pittsburgh compound B; coef: coefficient from linear regression model; pval: p-value

**Supplementary Table 5. Sensitivity analyses: Associations between serum metabolite concentrations and brain amyloid-β deposition, longitudinal changes in global brain WML burden, and rates of brain atrophy - BLSA**

|  | **Global DVR (amyloid-β deposition) – amyloid +ve sample** | | | | | | | | |
| --- | --- | --- | --- | --- | --- | --- | --- | --- | --- |
|  | **Total** | | | **Male** | | | **Female** | | |
|  | **coef** | **pval** | | **coef** | **pval** | | **coef** | **pval** | |
| 7α-Hydroxycholesterol | -1.631645 | 0.0277457 | | -1.837312 | 0.0576163 | | -0.941191 | 0.6143332 | |
| Chenodeoxycholic Acid | 2.89674 | 0.0904543 | | 3.240786 | 0.1699186 | | 4.337093 | 0.2603906 | |
| Cholic Acid | 3.984977 | 0.0246335 | | 4.654323 | 0.0603609 | | 2.457011 | 0.5357193 | |
|  | **Precuneus DVR (amyloid-β deposition) – amyloid +ve sample** | | | | | | | | |
|  | **Total** | | | **Male** | | | **Female** | | |
|  | **coef** | **pval** | | **coef** | **pval** | | **coef** | **pval** | |
| 7α-Hydroxycholesterol | -1.344883 | 0.0274872 | | -1.78109 | 0.0266959 | | 0.0752554 | 0.9585649 | |
| Chenodeoxycholic Acid | 2.398348 | 0.0884328 | | 2.700372 | 0.1776736 | | 4.769926 | 0.0964255 | |
| Cholic Acid | 2.471466 | 0.0966137 | | 2.909035 | 0.1775438 | | 2.164256 | 0.4778907 | |
|  | **White Matter Lesions (WML)** | | | | | | | | |
|  | **Total** | | | **Male** | | | **Female** | | |
|  | **coef** | **pval** | | **coef** | **pval** | | **coef** | **pval** | |
| 7α-Hydroxycholesterol | 0.0151573 | 0.085951 | | -0.0094945 | 0.42574 | | 0.0313322 | 0.0103825 | |
| Chenodeoxycholic Acid | -0.0004981 | 0.8921677 | | -0.0092819 | 0.0503062 | | 0.0048533 | 0.3455819 | |
| Cholic Acid | -0.0009591 | 0.7753811 | | -0.0011441 | 0.7666705 | | 0.0000979 | 0.9851934 | |
|  | **Brain Atrophy** | | | | | | | | |
|  | **Total** | | | **Male** | | | **Female** | | |
|  | **coef** | **pval** | **pval (FDR)** | **coef** | **pval** | **pval (FDR)** | **coef** | **pval** | **pval (FDR)** |
| Chenodeoxycholic Acid (Parietal gray matter) | 0.0346257 | 0.4237479 | 0.71334546 | 0.291119 | 0.0001555 | 0.00279861 | -0.1121444 | 0.0137629 | 0.11906992 |
| Chenodeoxycholic Acid (Precuneus) | 0.0194679 | 0.0988097 | 0.4853322 | 0.0947117 | 1.04E-06 | 0.00005613 | -0.0218086 | 0.1009093 | 0.30511338 |
| Cholic Acid (Parietal gray matter) | 0.0229564 | 0.5627652 | 0.78116325 | 0.2083698 | 0.0008666 | 0.01169843 | -0.1536291 | 0.0007058 | 0.03811246 |
| Cholic Acid (Precuneus) | 0.0162737 | 0.1322897 | 0.59530375 | 0.0699067 | 9.86E-06 | 0.00026634 | -0.0312286 | 0.019845 | 0.11906992 |

Sensitivity analyses after including statin use as a covariate; BLSA: Baltimore Longitudinal Study of Aging; WML: white matter lesions; coef: coefficient from linear regression model or mixed effects model; pval: p-value; FDR: False Discovery Rate (Benjamini-Hochberg) corrected p-value.

| **Supplementary Table 6. Characteristics of participants who received at least 2 BAS or LMT prescriptions with at least 1 year of follow-up after 2^nd^ prescription** | | | | | |
| --- | --- | --- | --- | --- | --- |
| **Variable** | **BAS users (N=3208)** | | **LMT users (N=23483)** | | **p-value** |
| Age at index date |  |  |  |  | <.001 ^1^ |
| . Mean, SD | 65.1 | (9.4) | 65.8 | (8.8) |  |
| . Median (min, max) | 64.1 | (50.0, 92.8) | 65.4 | (45.1, 96.7) |  |
| Age at index date, n (%) |  |  |  |  | <.001 ^2^ |
| . 45-50 | 0 | (0%) | 467 | (2%) |  |
| . 50-<60 | 1124 | (35%) | 6174 | (26%) |  |
| . 60-<70 | 1118 | (35%) | 9250 | (39%) |  |
| . 70-96.7 | 966 | (30%) | 7592 | (32%) |  |
| Patient’s sex, n (%) |  |  |  |  | <.001 ^2^ |
| . Male | 1083 | (34%) | 8977 | (38%) |  |
| . Female | 2125 | (66%) | 14506 | (62%) |  |
| Alcohol consumption, n (%) |  |  |  |  | <.001 ^2^ |
| . Ever | 2459 | (77%) | 19367 | (82%) |  |
| . Never | 428 | (13%) | 2597 | (11%) |  |
| . Missing | 321 | (10%) | 1519 | (6%) |  |
| Smoking status, n (%) |  |  |  |  | <.001 ^2^ |
| . Ever | 2464 | (77%) | 18761 | (80%) |  |
| . Never | 641 | (20%) | 4400 | (19%) |  |
| . Missing | 103 | (3%) | 322 | (1%) |  |
| BMI, n (%) |  |  |  |  | <.001 ^2^ |
| . Low/normal (<25) | 1143 | (36%) | 5384 | (23%) |  |
| . Overweight/ obese (>=25) | 1837 | (57%) | 17089 | (73%) |  |
| . Missing | 228 | (7%) | 1010 | (4%) |  |
| Statins use^3^, n (%) |  |  |  |  | <.001 ^2^ |
| . Yes | 828 | (26%) | 18804 | (80%) |  |
| . No | 2380 | (74%) | 4679 | (20%) |  |
| Metformin use^3^, n (%) |  |  |  |  | <.001 ^2^ |
| . Yes | 237 | (7%) | 3543 | (15%) |  |
| . No | 2971 | (93%) | 19940 | (85%) |  |
| Coronary Artery Disease^3^, n (%) |  |  |  |  | <.001 ^2^ |
| . Yes | 88 | (3%) | 1687 | (7%) |  |
| . No | 3120 | (97%) | 21796 | (93%) |  |
| Type 2 Diabetes^3^, n (%) |  |  |  |  | <.001 ^2^ |
| . Yes | 91 | (3%) | 1598 | (7%) |  |
| . No | 3117 | (97%) | 21885 | (93%) |  |
| Dyslipidemia^3^, n (%) |  |  |  |  | <.001 ^2^ |
| . Yes | 163 | (5%) | 5958 | (25%) |  |
| . No | 3045 | (95%) | 17525 | (75%) |  |
| Prior cancer diagnosis, n (%) |  |  |  |  | <.001 ^2^ |
| . Yes | 525 | (16%) | 1894 | (8%) |  |
| . No | 2683 | (84%) | 21589 | (92%) |  |
| Index year, n (%) |  |  |  |  | <.001 ^2^ |
| . 1995-2004 | 880 | (27%) | 5442 | (23%) |  |
| . 2005-2008 | 683 | (21%) | 8234 | (35%) |  |
| . 2009-2011 | 648 | (20%) | 5628 | (24%) |  |
| . 2012-2017 | 997 | (31%) | 4179 | (18%) |  |
| Registration year, n (%) |  |  |  |  | 0.045 ^2^ |
| . 1920-1970 | 397 | (12%) | 2741 | (12%) |  |
| . 1971-1980 | 429 | (13%) | 2864 | (12%) |  |
| . 1981-1990 | 804 | (25%) | 6016 | (26%) |  |
| . 1991-2000 | 952 | (30%) | 7470 | (32%) |  |
| . 2001-2016 | 626 | (20%) | 4392 | (19%) |  |
| Any dementia^4^, n (%) |  |  |  |  | 0.006 ^2^ |
| . Yes | 72 | (2%) | 737 | (3%) |  |
| . No | 3136 | (98%) | 22746 | (97%) |  |
| Alzheimer’s disease^4^, n (%) |  |  |  |  | 0.09 ^2^ |
| . Yes | 30 | (1%) | 302 | (1%) |  |
| . No | 3178 | (99%) | 23181 | (99%) |  |
| Vascular dementia^4^, n (%) |  |  |  |  | 0.47 ^2^ |
| . Yes | 31 | (1%) | 260 | (1%) |  |
| . No | 3177 | (99%) | 23223 | (99%) |  |
| Other dementia, not otherwise specified^4^, n (%) |  |  |  |  | 0.01 ^2^ |
| . Yes | 11 | (0%) | 175 | (1%) |  |
| . No | 3197 | (100%) | 23308 | (99%) |  |
| 1. Wilcoxon rank-sum test 2. Chi-squared test 3. 1 year prior to index date 4. During study follow-up | | | | | |

**Supplementary Table 7. Characteristics of participants with incident dementia event during follow-up**

| **Variable** | **Alzheimer’s**  **disease (N=332)** | | **Vascular**  **dementia (N=291)** | | **Other dementia,**  **Not otherwise specified (N=186)** | |
| --- | --- | --- | --- | --- | --- | --- |
| Age at index date |  |  |  |  |  |  |
| Mean, SD | 72.3 | (6.8) | 72.9 | (6.9) | 73.3 | (7.3) |
| Median (min, max) | 72.4 | (51.6, 88.8) | 73.4 | (52.2, 91.5) | 73.7 | (55.9, 92.0) |
| Age at index date, n (%) |  |  |  |  |  |  |
| 50-<60 | 12 | (4%) | 11 | (4%) | 7 | (4%) |
| 60-<70 | 100 | (30%) | 77 | (26%) | 58 | (31%) |
| 70 or older | 220 | (66%) | 203 | (70%) | 121 | (65%) |
| Patient sex, n (%) |  |  |  |  |  |  |
| male | 97 | (29%) | 104 | (36%) | 64 | (34%) |
| female | 235 | (71%) | 187 | (64%) | 122 | (66%) |
| Alcohol consumption, n (%) |  |  |  |  |  |  |
| ever | 264 | (80%) | 246 | (85%) | 143 | (77%) |
| never | 44 | (13%) | 32 | (11%) | 24 | (13%) |
| missing | 24 | (7%) | 13 | (4%) | 19 | (10%) |
| Smoking status, n (%) |  |  |  |  |  |  |
| ever | 274 | (83%) | 246 | (85%) | 146 | (78%) |
| never | 51 | (15%) | 37 | (13%) | 32 | (17%) |
| missing | 7 | (2%) | 8 | (3%) | 8 | (4%) |
| BMI, n (%) |  |  |  |  |  |  |
| Low/normal (<25) | 122 | (37%) | 91 | (31%) | 60 | (32%) |
| Overweight/ obese (≥25) | 195 | (59%) | 190 | (65%) | 114 | (61%) |
| Missing | 15 | (5%) | 10 | (3%) | 12 | (6%) |
| Statins use^1^, n (%) |  |  |  |  |  |  |
| Yes | 256 | (77%) | 217 | (75%) | 138 | (74%) |
| No | 76 | (23%) | 74 | (25%) | 48 | (26%) |
| Metformin use^1^, n (%) |  |  |  |  |  |  |
| Yes | 31 | (9%) | 42 | (14%) | 22 | (12%) |
| No | 301 | (91%) | 249 | (86%) | 164 | (88%) |
| Coronary Artery Disease^1^, n (%) |  |  |  |  |  |  |
| Yes | 21 | (6%) | 32 | (11%) | 14 | (8%) |
| No | 311 | (94%) | 259 | (89%) | 172 | (92%) |
| Type 2 Diabetes^1^, n (%) |  |  |  |  |  |  |
| Yes | 16 | (5%) | 23 | (8%) | 17 | (9%) |
| No | 316 | (95%) | 268 | (92%) | 169 | (91%) |
| Dyslipidemia^1^, n (%) |  |  |  |  |  |  |
| Yes | 92 | (28%) | 71 | (24%) | 50 | (27%) |
| No | 240 | (72%) | 220 | (76%) | 136 | (73%) |
| Prior cancer diagnosis, n (%) |  |  |  |  |  |  |
| Yes | 37 | (11%) | 30 | (10%) | 21 | (11%) |
| No | 295 | (89%) | 261 | (90%) | 165 | (89%) |
| Index year, n (%) |  |  |  |  |  |  |
| 1995-2004 | 118 | (36%) | 96 | (33%) | 71 | (38%) |
| 2005-2008 | 139 | (42%) | 121 | (42%) | 74 | (40%) |
| 2009-2011 | 56 | (17%) | 60 | (21%) | 34 | (18%) |
| 2012-2017 | 19 | (6%) | 14 | (5%) | 7 | (4%) |
| Registration year, n (%) |  |  |  |  |  |  |
| 1920-1970 | 47 | (14%) | 53 | (18%) | 36 | (19%) |
| 1971-1980 | 50 | (15%) | 28 | (10%) | 22 | (12%) |
| 1981-1990 | 77 | (23%) | 83 | (29%) | 55 | (30%) |
| 1991-2000 | 115 | (35%) | 87 | (30%) | 56 | (30%) |
| 2001-2016 | 43 | (13%) | 40 | (14%) | 17 | (9%) |
| 1. 1 year prior to index date | | | | | | |

**Supplementary Table 8. Demographic characteristics of BLSA autopsy sample**

|  | AD | CON |
| --- | --- | --- |
| Sample size | 16 | 13 |
| Age at death, mean (SD) | 87.4 (9.5) | 82.4 (11.5) |
| Male Sex, n (% male) | 8 (50.0) | 10 (76.9) |
| White Race, n (% white) | 16 (100.0) | 12 (92.3) |
| APOE4, n (% e4) | 4 (25.0) | 3 (23.1) |
| PMI, mean (SD) | 14.2 (5.7) | 16.8 (6.0) |

AD: Alzheimer’s disease; CON: control; APOE4: Apolipoprotein E allele epsilon 4; PMI: postmortem interval (hrs)

**Supplementary Table 9. Differences in brain primary bile acid concentrations between AD and CON**

|  | ITG | | MFG | | CB | |
| --- | --- | --- | --- | --- | --- | --- |
|  | coef | pval | coef | pval | coef | pval |
| Chenodeoxycholic acid | 0.772 | 0.074 | 0.632 | 0.109 | * | 0.026 |
| Cholic acid | 0.244 | 0.092 | 0.230 | 0.085 | 0.172 | 0.133 |

coef: coefficient for disease (AD vs CON) from the tobit model including mean-centered age and sex where the lower limit is set as the metabolite specific limit of detection (LOD); pval: p-value

* In the CON sample in the CB, all concentrations were below LOD; we therefore tested for differences in the number of concentrations below LOD comparing AD to CON using the chi2 test and present the associated p-value

**Supplementary Table 10. Differences in brain BA receptor gene expression between AD and CON**

[see attached xls table: Supplementary_Table_10.xlsx]
