# Supplementary material for: Bile acid synthesis, modulation, and dementia: A metabolomic, transcriptomic, and pharmacoepidemiologic study
Source: PLoS Med. 2021 May 27;18(5):e1003615. doi: 10.1371/journal.pmed.1003615 (PMC8158920; doi:10.1371/journal.pmed.1003615)
Supplement: S3 Table — (A) Demographic characteristics of BLSA-NI sample. APOE4, e4 allele of the Apolipoprotein E gene; BLSA, Baltimore Longitudinal Study of Aging; MRI, magnetic resonance imaging; NI, neuroimaging; PiB, Pittsburgh compound B; SD, standard deviation; WML, white matter lesion. (B) Demographic characteristics of ADNI sample. ADNI, Alzheimer’s Disease Neuroimaging Initiative; MRI, magnetic resonance imaging; NI, neuroimaging; SD, standard deviation; WML, white matter lesion (DOCX) [file pmed.1003615.s005.docx]

**Supplementary Table 3A. Demographic characteristics of BLSA-NI sample**

|  | PiB Analyses | Brain atrophy/ WML analyses |
| --- | --- | --- |
| Sample size | 141 | 134 |
| Age, mean (SD) | 76.1 (8.5) | 76.1 (8.5) |
| Male Sex, n (% male) | 66 (46.8) | 62 (46.3) |
| White Race, n (% white) | 107 (75.9) | 102 (76.1) |
| Amyloid +ve, n (% +ve) | 36 (25.5) | - |
| APOE4, n (% e4) | 36 (27.1) | 35 (26.3) |
| Longitudinal MRI visits, n | - | 332 |
| Follow-up MRI visits, mean (SD) | - | 2.5 (1.5) |

BLSA: Baltimore Longitudinal Study of Aging; NI: Neuroimaging; WML: White Matter Lesions; PiB: Pittsburgh compound B; MRI: Magnetic Resonance Imaging; APOE4: e4 allele of the Apolipoprotein E gene; SD: standard deviation

**Supplementary Table 3B. Demographic characteristics of ADNI sample**

|  | Brain atrophy/ WML analyses |
| --- | --- |
| Sample size | 1666 |
| Age, mean (SD) | 73.8 (7.2) |
| Male Sex, n (% male) | 918 (55.1) |
| White Race, n (% white) | 1538 (92.5) |
| Longitudinal MRI visits, n | 8686 |
| Follow-up MRI visits, mean (SD) | 5.2 (2.5) |

ADNI: Alzheimer’s Disease Neuroimaging Initiative; NI: Neuroimaging; WML: White Matter Lesions; MRI: Magnetic Resonance Imaging; SD: standard deviation
