# Supplementary material for: Bile acid synthesis, modulation, and dementia: A metabolomic, transcriptomic, and pharmacoepidemiologic study
Source: PLoS Med. 2021 May 27;18(5):e1003615. doi: 10.1371/journal.pmed.1003615 (PMC8158920; doi:10.1371/journal.pmed.1003615)
Supplement: S4 Table — coef, coefficient from linear regression model; PiB, Pittsburgh compound B; pval, p-value. (DOCX) [file pmed.1003615.s006.docx]

**Supplementary Table 4. Associations between serum metabolite concentrations and PiB/ amyloid status**

|  | Total | | Male | | Female | |
| --- | --- | --- | --- | --- | --- | --- |
|  | coef | pval | coef | pval | coef | pval |
| 7α-hydroxycholesterol | -0.205 | 0.127 | -0.21 | 0.246 | -0.127 | 0.536 |
| Chenodeoxycholic acid | 0.002 | 0.996 | 0.236 | 0.598 | -0.186 | 0.669 |
| Cholic acid | 0.35 | 0.262 | 0.364 | 0.45 | 0.388 | 0.372 |

PiB: Pittsburgh compound B; coef: coefficient from linear regression model; pval: p-value
