# Supplementary material for: Bile acid synthesis, modulation, and dementia: A metabolomic, transcriptomic, and pharmacoepidemiologic study
Source: PLoS Med. 2021 May 27;18(5):e1003615. doi: 10.1371/journal.pmed.1003615 (PMC8158920; doi:10.1371/journal.pmed.1003615)
Supplement: S5 Table — Sensitivity analyses after including statin use as a covariate. BLSA, Baltimore Longitudinal Study of Aging; coef, coefficient from linear regression model or mixed effects model; FDR, false discovery rate (Benjamini–Hochberg) corrected p-value; pval, p-value; WML, white matter lesion. (DOCX) [file pmed.1003615.s007.docx]

**Supplementary Table 5. Sensitivity analyses: Associations between serum metabolite concentrations and brain amyloid-β deposition, longitudinal changes in global brain WML burden, and rates of brain atrophy - BLSA**

|  | **Global DVR (amyloid-β deposition) – amyloid +ve sample** | | | | | | | | |
| --- | --- | --- | --- | --- | --- | --- | --- | --- | --- |
|  | **Total** | | | **Male** | | | **Female** | | |
|  | **coef** | **pval** | | **coef** | **pval** | | **coef** | **pval** | |
| 7α-Hydroxycholesterol | -1.631645 | 0.0277457 | | -1.837312 | 0.0576163 | | -0.941191 | 0.6143332 | |
| Chenodeoxycholic Acid | 2.89674 | 0.0904543 | | 3.240786 | 0.1699186 | | 4.337093 | 0.2603906 | |
| Cholic Acid | 3.984977 | 0.0246335 | | 4.654323 | 0.0603609 | | 2.457011 | 0.5357193 | |
|  | **Precuneus DVR (amyloid-β deposition) – amyloid +ve sample** | | | | | | | | |
|  | **Total** | | | **Male** | | | **Female** | | |
|  | **coef** | **pval** | | **coef** | **pval** | | **coef** | **pval** | |
| 7α-Hydroxycholesterol | -1.344883 | 0.0274872 | | -1.78109 | 0.0266959 | | 0.0752554 | 0.9585649 | |
| Chenodeoxycholic Acid | 2.398348 | 0.0884328 | | 2.700372 | 0.1776736 | | 4.769926 | 0.0964255 | |
| Cholic Acid | 2.471466 | 0.0966137 | | 2.909035 | 0.1775438 | | 2.164256 | 0.4778907 | |
|  | **White Matter Lesions (WML)** | | | | | | | | |
|  | **Total** | | | **Male** | | | **Female** | | |
|  | **coef** | **pval** | | **coef** | **pval** | | **coef** | **pval** | |
| 7α-Hydroxycholesterol | 0.0151573 | 0.085951 | | -0.0094945 | 0.42574 | | 0.0313322 | 0.0103825 | |
| Chenodeoxycholic Acid | -0.0004981 | 0.8921677 | | -0.0092819 | 0.0503062 | | 0.0048533 | 0.3455819 | |
| Cholic Acid | -0.0009591 | 0.7753811 | | -0.0011441 | 0.7666705 | | 0.0000979 | 0.9851934 | |
|  | **Brain Atrophy** | | | | | | | | |
|  | **Total** | | | **Male** | | | **Female** | | |
|  | **coef** | **pval** | **pval (FDR)** | **coef** | **pval** | **pval (FDR)** | **coef** | **pval** | **pval (FDR)** |
| Chenodeoxycholic Acid (Parietal gray matter) | 0.0346257 | 0.4237479 | 0.71334546 | 0.291119 | 0.0001555 | 0.00279861 | -0.1121444 | 0.0137629 | 0.11906992 |
| Chenodeoxycholic Acid (Precuneus) | 0.0194679 | 0.0988097 | 0.4853322 | 0.0947117 | 1.04E-06 | 0.00005613 | -0.0218086 | 0.1009093 | 0.30511338 |
| Cholic Acid (Parietal gray matter) | 0.0229564 | 0.5627652 | 0.78116325 | 0.2083698 | 0.0008666 | 0.01169843 | -0.1536291 | 0.0007058 | 0.03811246 |
| Cholic Acid (Precuneus) | 0.0162737 | 0.1322897 | 0.59530375 | 0.0699067 | 9.86E-06 | 0.00026634 | -0.0312286 | 0.019845 | 0.11906992 |

Sensitivity analyses after including statin use as a covariate; BLSA: Baltimore Longitudinal Study of Aging; WML: white matter lesions; coef: coefficient from linear regression model or mixed effects model; pval: p-value; FDR: False Discovery Rate (Benjamini-Hochberg) corrected p-value.
