# Supplementary material for: Bile acid synthesis, modulation, and dementia: A metabolomic, transcriptomic, and pharmacoepidemiologic study
Source: PLoS Med. 2021 May 27;18(5):e1003615. doi: 10.1371/journal.pmed.1003615 (PMC8158920; doi:10.1371/journal.pmed.1003615)
Supplement: S6 Table — Wilcoxon rank-sum test. 1 Chi-squared test. 2 1 year prior to index date. 3 During study follow-up. BAS, bile acid sequestrants; LMT, lipid-modifying therapies. (DOCX) [file pmed.1003615.s008.docx]

| **Supplementary Table 6. Characteristics of participants who received at least 2 BAS or LMT prescriptions with at least 1 year of follow-up after 2^nd^ prescription** | | | | | |
| --- | --- | --- | --- | --- | --- |
| **Variable** | **BAS users (N=3208)** | | **LMT users (N=23483)** | | **p-value** |
| Age at index date |  |  |  |  | <.001 ^1^ |
| . Mean, SD | 65.1 | (9.4) | 65.8 | (8.8) |  |
| . Median (min, max) | 64.1 | (50.0, 92.8) | 65.4 | (45.1, 96.7) |  |
| Age at index date, n (%) |  |  |  |  | <.001 ^2^ |
| . 45-50 | 0 | (0%) | 467 | (2%) |  |
| . 50-<60 | 1124 | (35%) | 6174 | (26%) |  |
| . 60-<70 | 1118 | (35%) | 9250 | (39%) |  |
| . 70-96.7 | 966 | (30%) | 7592 | (32%) |  |
| Patient’s sex, n (%) |  |  |  |  | <.001 ^2^ |
| . Male | 1083 | (34%) | 8977 | (38%) |  |
| . Female | 2125 | (66%) | 14506 | (62%) |  |
| Alcohol consumption, n (%) |  |  |  |  | <.001 ^2^ |
| . Ever | 2459 | (77%) | 19367 | (82%) |  |
| . Never | 428 | (13%) | 2597 | (11%) |  |
| . Missing | 321 | (10%) | 1519 | (6%) |  |
| Smoking status, n (%) |  |  |  |  | <.001 ^2^ |
| . Ever | 2464 | (77%) | 18761 | (80%) |  |
| . Never | 641 | (20%) | 4400 | (19%) |  |
| . Missing | 103 | (3%) | 322 | (1%) |  |
| BMI, n (%) |  |  |  |  | <.001 ^2^ |
| . Low/normal (<25) | 1143 | (36%) | 5384 | (23%) |  |
| . Overweight/ obese (>=25) | 1837 | (57%) | 17089 | (73%) |  |
| . Missing | 228 | (7%) | 1010 | (4%) |  |
| Statins use^3^, n (%) |  |  |  |  | <.001 ^2^ |
| . Yes | 828 | (26%) | 18804 | (80%) |  |
| . No | 2380 | (74%) | 4679 | (20%) |  |
| Metformin use^3^, n (%) |  |  |  |  | <.001 ^2^ |
| . Yes | 237 | (7%) | 3543 | (15%) |  |
| . No | 2971 | (93%) | 19940 | (85%) |  |
| Coronary Artery Disease^3^, n (%) |  |  |  |  | <.001 ^2^ |
| . Yes | 88 | (3%) | 1687 | (7%) |  |
| . No | 3120 | (97%) | 21796 | (93%) |  |
| Type 2 Diabetes^3^, n (%) |  |  |  |  | <.001 ^2^ |
| . Yes | 91 | (3%) | 1598 | (7%) |  |
| . No | 3117 | (97%) | 21885 | (93%) |  |
| Dyslipidemia^3^, n (%) |  |  |  |  | <.001 ^2^ |
| . Yes | 163 | (5%) | 5958 | (25%) |  |
| . No | 3045 | (95%) | 17525 | (75%) |  |
| Prior cancer diagnosis, n (%) |  |  |  |  | <.001 ^2^ |
| . Yes | 525 | (16%) | 1894 | (8%) |  |
| . No | 2683 | (84%) | 21589 | (92%) |  |
| Index year, n (%) |  |  |  |  | <.001 ^2^ |
| . 1995-2004 | 880 | (27%) | 5442 | (23%) |  |
| . 2005-2008 | 683 | (21%) | 8234 | (35%) |  |
| . 2009-2011 | 648 | (20%) | 5628 | (24%) |  |
| . 2012-2017 | 997 | (31%) | 4179 | (18%) |  |
| Registration year, n (%) |  |  |  |  | 0.045 ^2^ |
| . 1920-1970 | 397 | (12%) | 2741 | (12%) |  |
| . 1971-1980 | 429 | (13%) | 2864 | (12%) |  |
| . 1981-1990 | 804 | (25%) | 6016 | (26%) |  |
| . 1991-2000 | 952 | (30%) | 7470 | (32%) |  |
| . 2001-2016 | 626 | (20%) | 4392 | (19%) |  |
| Any dementia^4^, n (%) |  |  |  |  | 0.006 ^2^ |
| . Yes | 72 | (2%) | 737 | (3%) |  |
| . No | 3136 | (98%) | 22746 | (97%) |  |
| Alzheimer’s disease^4^, n (%) |  |  |  |  | 0.09 ^2^ |
| . Yes | 30 | (1%) | 302 | (1%) |  |
| . No | 3178 | (99%) | 23181 | (99%) |  |
| Vascular dementia^4^, n (%) |  |  |  |  | 0.47 ^2^ |
| . Yes | 31 | (1%) | 260 | (1%) |  |
| . No | 3177 | (99%) | 23223 | (99%) |  |
| Other dementia, not otherwise specified^4^, n (%) |  |  |  |  | 0.01 ^2^ |
| . Yes | 11 | (0%) | 175 | (1%) |  |
| . No | 3197 | (100%) | 23308 | (99%) |  |
| 1. Wilcoxon rank-sum test 2. Chi-squared test 3. 1 year prior to index date 4. During study follow-up | | | | | |
