# Supplementary material for: Bile acid synthesis, modulation, and dementia: A metabolomic, transcriptomic, and pharmacoepidemiologic study
Source: PLoS Med. 2021 May 27;18(5):e1003615. doi: 10.1371/journal.pmed.1003615 (PMC8158920; doi:10.1371/journal.pmed.1003615)
Supplement: S7 Table — 1 1 year prior to index date. (DOCX) [file pmed.1003615.s009.docx]

**Supplementary Table 7. Characteristics of participants with incident dementia event during follow-up**

| **Variable** | **Alzheimer’s**  **disease (N=332)** | | **Vascular**  **dementia (N=291)** | | **Other dementia,**  **Not otherwise specified (N=186)** | |
| --- | --- | --- | --- | --- | --- | --- |
| Age at index date |  |  |  |  |  |  |
| Mean, SD | 72.3 | (6.8) | 72.9 | (6.9) | 73.3 | (7.3) |
| Median (min, max) | 72.4 | (51.6, 88.8) | 73.4 | (52.2, 91.5) | 73.7 | (55.9, 92.0) |
| Age at index date, n (%) |  |  |  |  |  |  |
| 50-<60 | 12 | (4%) | 11 | (4%) | 7 | (4%) |
| 60-<70 | 100 | (30%) | 77 | (26%) | 58 | (31%) |
| 70 or older | 220 | (66%) | 203 | (70%) | 121 | (65%) |
| Patient sex, n (%) |  |  |  |  |  |  |
| male | 97 | (29%) | 104 | (36%) | 64 | (34%) |
| female | 235 | (71%) | 187 | (64%) | 122 | (66%) |
| Alcohol consumption, n (%) |  |  |  |  |  |  |
| ever | 264 | (80%) | 246 | (85%) | 143 | (77%) |
| never | 44 | (13%) | 32 | (11%) | 24 | (13%) |
| missing | 24 | (7%) | 13 | (4%) | 19 | (10%) |
| Smoking status, n (%) |  |  |  |  |  |  |
| ever | 274 | (83%) | 246 | (85%) | 146 | (78%) |
| never | 51 | (15%) | 37 | (13%) | 32 | (17%) |
| missing | 7 | (2%) | 8 | (3%) | 8 | (4%) |
| BMI, n (%) |  |  |  |  |  |  |
| Low/normal (<25) | 122 | (37%) | 91 | (31%) | 60 | (32%) |
| Overweight/ obese (≥25) | 195 | (59%) | 190 | (65%) | 114 | (61%) |
| Missing | 15 | (5%) | 10 | (3%) | 12 | (6%) |
| Statins use^1^, n (%) |  |  |  |  |  |  |
| Yes | 256 | (77%) | 217 | (75%) | 138 | (74%) |
| No | 76 | (23%) | 74 | (25%) | 48 | (26%) |
| Metformin use^1^, n (%) |  |  |  |  |  |  |
| Yes | 31 | (9%) | 42 | (14%) | 22 | (12%) |
| No | 301 | (91%) | 249 | (86%) | 164 | (88%) |
| Coronary Artery Disease^1^, n (%) |  |  |  |  |  |  |
| Yes | 21 | (6%) | 32 | (11%) | 14 | (8%) |
| No | 311 | (94%) | 259 | (89%) | 172 | (92%) |
| Type 2 Diabetes^1^, n (%) |  |  |  |  |  |  |
| Yes | 16 | (5%) | 23 | (8%) | 17 | (9%) |
| No | 316 | (95%) | 268 | (92%) | 169 | (91%) |
| Dyslipidemia^1^, n (%) |  |  |  |  |  |  |
| Yes | 92 | (28%) | 71 | (24%) | 50 | (27%) |
| No | 240 | (72%) | 220 | (76%) | 136 | (73%) |
| Prior cancer diagnosis, n (%) |  |  |  |  |  |  |
| Yes | 37 | (11%) | 30 | (10%) | 21 | (11%) |
| No | 295 | (89%) | 261 | (90%) | 165 | (89%) |
| Index year, n (%) |  |  |  |  |  |  |
| 1995-2004 | 118 | (36%) | 96 | (33%) | 71 | (38%) |
| 2005-2008 | 139 | (42%) | 121 | (42%) | 74 | (40%) |
| 2009-2011 | 56 | (17%) | 60 | (21%) | 34 | (18%) |
| 2012-2017 | 19 | (6%) | 14 | (5%) | 7 | (4%) |
| Registration year, n (%) |  |  |  |  |  |  |
| 1920-1970 | 47 | (14%) | 53 | (18%) | 36 | (19%) |
| 1971-1980 | 50 | (15%) | 28 | (10%) | 22 | (12%) |
| 1981-1990 | 77 | (23%) | 83 | (29%) | 55 | (30%) |
| 1991-2000 | 115 | (35%) | 87 | (30%) | 56 | (30%) |
| 2001-2016 | 43 | (13%) | 40 | (14%) | 17 | (9%) |
| 1. 1 year prior to index date | | | | | | |
