# Supplementary material for: Bile acid synthesis, modulation, and dementia: A metabolomic, transcriptomic, and pharmacoepidemiologic study
Source: PLoS Med. 2021 May 27;18(5):e1003615. doi: 10.1371/journal.pmed.1003615 (PMC8158920; doi:10.1371/journal.pmed.1003615)
Supplement: S8 Table — AD, Alzheimer disease; APOE4, apolipoprotein E allele epsilon 4; BLSA, Baltimore Longitudinal Study of Aging; CON, control; PMI, postmortem interval (hours). (DOCX) [file pmed.1003615.s010.docx]

**Supplementary Table 8. Demographic characteristics of BLSA autopsy sample**

|  | AD | CON |
| --- | --- | --- |
| Sample size | 16 | 13 |
| Age at death, mean (SD) | 87.4 (9.5) | 82.4 (11.5) |
| Male Sex, n (% male) | 8 (50.0) | 10 (76.9) |
| White Race, n (% white) | 16 (100.0) | 12 (92.3) |
| APOE4, n (% e4) | 4 (25.0) | 3 (23.1) |
| PMI, mean (SD) | 14.2 (5.7) | 16.8 (6.0) |

AD: Alzheimer’s disease; CON: control; APOE4: Apolipoprotein E allele epsilon 4; PMI: postmortem interval (hrs)
