# Supplementary material for: Bile acid synthesis, modulation, and dementia: A metabolomic, transcriptomic, and pharmacoepidemiologic study
Source: PLoS Med. 2021 May 27;18(5):e1003615. doi: 10.1371/journal.pmed.1003615 (PMC8158920; doi:10.1371/journal.pmed.1003615)
Supplement: S9 Table — * In the CON sample in the CB, all concentrations were below LOD; we therefore tested for differences in the number of concentrations below LOD comparing AD to CON using the chi-squared test and present the associated p-value. AD, Alzheimer disease; BA, bile acid; coef, coefficient for disease (AD vs. CON) from the tobit model including mean-centered age and sex where the lower limit is set as the metabolite specific LOD; CON, control; LOD, limit of detection; pval, p-value. (DOCX) [file pmed.1003615.s011.docx]

**Supplementary Table 9. Differences in brain primary bile acid concentrations between AD and CON**

|  | ITG | | MFG | | CB | |
| --- | --- | --- | --- | --- | --- | --- |
|  | coef | pval | coef | pval | coef | pval |
| Chenodeoxycholic acid | 0.772 | 0.074 | 0.632 | 0.109 | * | 0.026 |
| Cholic acid | 0.244 | 0.092 | 0.230 | 0.085 | 0.172 | 0.133 |

coef: coefficient for disease (AD vs CON) from the tobit model including mean-centered age and sex where the lower limit is set as the metabolite specific limit of detection (LOD); pval: p-value

* In the CON sample in the CB, all concentrations were below LOD; we therefore tested for differences in the number of concentrations below LOD comparing AD to CON using the chi2 test and present the associated p-value
