# Supplementary material for: Bile acid synthesis, modulation, and dementia: A metabolomic, transcriptomic, and pharmacoepidemiologic study
Source: PLoS Med. 2021 May 27;18(5):e1003615. doi: 10.1371/journal.pmed.1003615 (PMC8158920; doi:10.1371/journal.pmed.1003615)
Supplement: Attachment — Submitted filename: Summary_response_9.2.20.docx [file pmed.1003615.s013.docx]

Note: reviewer comments are indicated in black text. Our response to reviewer recommendations are indicated in red text immediately below each comment. We have additionally included relevant text from the revised manuscript below each response in *red italics.*

**Editor Requests**

*We'd suggest revising the title according to PLOS Medicine's style; this should have the initial phrase outlining the study question and then the study design(s) in the second phrase after a colon (eg, (: "randomized controlled trial," "A retrospective study," "A modelling study," etc.)

We have revised the study title to: **“**Bile acid synthesis and modulation are associated with brain amyloid deposition, white matter lesions, neurodegeneration and risk of vascular dementia: a metabolomic, neuroimaging, pharmacoepidemiologic and transcriptomic analysis”

*In the last sentence of the Abstract Methods and Findings section, please describe the main limitation(s) of the study's methodology.

We have added the main limitation to the Abstract Methods and Findings section.

*Limitations of our study include the relatively small sample sizes in the BLSA neuroimaging and autopsy cohorts as well as likely inaccuracies in the clinical diagnosis of dementia subtypes in primary care settings as reflected in CPRD outcomes. Our findings merit confirmation in other independent studies.*

*At this stage, we ask that you include a short, non-technical Author Summary of your research to make findings accessible to a wide audience that includes both scientists and non-scientists. The Author Summary should immediately follow the Abstract in your revised manuscript. This text is subject to editorial change and should be distinct from the scientific abstract. Please see our author guidelines for more information: <https://journals.plos.org/plosmedicine/s/revising-your-manuscript#loc-author-summary>

We added a short author-summary immediately following the Abstract.

*Please ensure the Methods section states whether the analysis plan followed in this paper for the 3 different studies was set out prospectively (ie prior to collection of data). Please state this (either way) early in the Methods section.

We have indicated in the Methods section that the analysis plan was not set out prospectively because all research participant data used in the study are derived from long-running longitudinal cohorts.

*All three cohorts were long-running and prospectively followed to help address broad questions related to aging and disease. Specific analyses addressing focused hypotheses described herein were not included in prospective analysis plans in the original study protocols for these cohorts.*

a) If a prospective analysis plan (from your funding proposal, IRB or other ethics committee submission, study protocol, or other planning document written before analyzing the data) was used in designing the study, please include the relevant prospectively written document with your revised manuscript as a Supporting Information file to be published alongside your study, and cite it in the Methods section. A legend for this file should be included at the end of your manuscript.

All the study cohorts used in the study were developed to answer broad questions related to aging and disease. Our specific analyses were not included in a prospective analysis plan.

b) If no such document exists, please make sure that the Methods section transparently describes when analyses were planned, and when/why any data-driven changes to analyses took place.

We have added the timeline for specific analyses to the Methods section. For each step of the analysis, we indicate in the Statistical Analysis section when the analyses were planned and modified. See an example below:

*Step 1 of the analytic plan using BLSA data was developed conceptually in January 2018 prior to starting analyses in June 2019. The inclusion of ADNI bile acid and neuroimaging data to validate significant BLSA BA results as well as sensitivity analyses (i.e. adding statin as a covariate) were performed in June 2020 in response to reviewer recommendations.*

c) In either case, changes in the analysis-- including those made in response to peer review comments-- should be identified as such in the Methods section of the paper, with rationale.

We have indicated any changes to the original analyses to the Methods section and indicated that those were made in response to peer review comments in the Statistical Analysis section for each step.

*Referencing callouts should ideally be sequential numerals in square brackets (ie [1], [2] etc). If referencing software was used then this should be fairly quick and easy.

We have edited reference format to square brackets.

*Please note the comments from one reviewer that the conclusions drawn in the paper should be more cautious, given the possible risks of multiple testing and the potential for false-positive findings (ie that some analyses may not survive correction for multiple testing).

We have more clearly indicated adjustment for multiple comparisons in the Methods section and have edited the Discussion section to more cautiously interpret our results. We have explicitly indicated in the Limitations section the risks of multiple testing and the potential for false-positive findings. In this context, it is also important to note that our revised manuscript now includes validation analyses in an independent cohort of participants with neuroimaging outcomes - i.e. ADNI - as well as single cell RNA-Seq data for brain gene expression analyses. Both these analyses now include FDR-adjustment to control for multiple comparisons.

**All three reviewers have commented on the below points which we address together.**

1) Address limitations due to small sample size and lack of confirmatory analyses in an independent cohort

In the revision, we have significantly expanded analyses to include two additional studies. First, we have included analyses of serum BA levels and neuroimaging outcomes from the Alzheimer’s Disease Neuroimaging Initiative (ADNI) to validate our index findings in BLSA. These analyses include 1,666 ADNI participants with serum BA data and an average of 5.6 longitudinal MRI visits (8686 visits in total). Our index BLSA results indicated a sex-specific association between BA and brain atrophy; we validated these findings in ADNI within similar brain regions. We were thus able to confirm our findings from the BLSA in an independent cohort that was demographically distinct. This validation provides further evidence for the sex specific association between bile acids and neuropathologic changes associated with dementia.

Second, we have included single-cell RNA-sequencing (scRNA-Seq) data from 46 Religious Orders Study and the Rush Memory and Aging Project (ROSMAP) participants. This included data across 8 major brain cell types in the aged dorsolateral prefrontal cortex including inhibitory neurons, excitatory neurons, astrocytes, oligodendrocytes, microglia, oligodendrocyte progenitor cells, endothelial cells, and pericytes. This significantly expanded gene expression dataset included multiple BA receptor genes and allowed us to derive a comprehensive understanding of changes in gene expression related to alterations in brain BA signaling in dementia. The larger sample size also enabled us to analyze sex-specific differences in BA receptor gene expression in AD.

2) Expand on rationale for sex specific analyses and provide more detailed discussion of sex-specific findings

We have expanded on our rationale for the *a priori* decision to explore sex-specific associations. We have added text to the introduction providing our justification for sex-stratified analyses and have referenced prior work to provide additional rationale for these analyses.

*Given prior evidence suggesting sex-specific differences in the serum lipidome as well as in the association between lipid levels and dementia risk [6, 7], we performed sex-stratified analyses to test the relationship between cholesterol catabolism and dementia.*

We have additionally expanded the Discussion section to more comprehensively discuss plausible mechanisms/ pathways explaining the observed sex-specific associations.

*While we have not addressed the precise mechanisms underlying sex-specific associations between BA metabolism and dementia pathogenesis, prior evidence suggests important sex differences in lipid metabolism that impact risk of cardiovascular disease [42, 43]. These effects extend to differences in cholesterol catabolism and sex-specific responsiveness to therapeutic lowering of cholesterol levels by increasing its conversion to BAs. Animal studies have also shown sex specific differences in bile acid homeostasis during aging and suggest that these may be mediated by differences in expression of BA transporters as well as CYP7A1, the rate-limiting enzyme in BA synthesis [44].*

**Reviewer 1**

1. It is summarized in the abstract that "We found that lower serum concentrations of 7α-OHC, CA and CDCA were associated with higher brain amyloid deposition, faster WML accumulation and faster brain atrophy in males. Opposite effects were observed in females". However, the actual data presented in Table 1 suggests that this description might be a simplification that possibly mischaracterizes the specifics. For example, it appears that no statistically-significant correlations between 7α-OHC/CA/CDCA and amyloid deposition was found for females, as was the case for CA/CDCA and amyloid deposition for males. Moreover, the Results section then states that "We observed no significant associations in the female only-sample" (line 298). The specific findings might thus be presented more precisely in the abstract.

We have updated the abstract to clarify the sex-specific findings as associations identified mainly in males.

*We found that lower serum concentrations of 7α-OHC, CA and CDCA were associated with higher brain amyloid deposition, faster WML accumulation and faster brain atrophy mainly in males in BLSA. We confirmed that lower serum concentration of CA and CDCA were associated with faster brain atrophy in males using data from ADNI.*

2. Perhaps of greater concern is that when analyzing all subjects (male+female) in Step 1, it appears that only 7α-OHC shows statistically-significant correlations with amyloid deposition/brain atrophy, with the actual BAs (CA/CDCA) exhibiting no correlations at all. The BA correlations appear only when considering a male/female sex-specific stratification of the cohort.

As such, it might be clarified whether this sex-specific analysis was *a priori* defined in the initial study design from some theoretical motivation/prior work, or if it was a post-hoc discovery. This is of interest due to the relatively small sample size (N=134/141) involved in Step 1, and also previous similar cited work on AD [citation 8] not automatically considering sex-based stratification.

We have added analyses of a substantially larger sample from ADNI to validate index findings of associations between BAs and brain atrophy in BLSA. We have also edited the paper to more precisely articulate the sex-specific findings.

Additionally, the decision to explore sex-specific associations was made *a priori*. We have added text to the introduction providing our justification for sex-stratified analyses and have referenced prior work to provide additional rationale for these analyses.

*Given prior evidence suggesting sex-specific differences in the serum lipidome as well as in the association between lipid levels and dementia risk [6, 7], we performed sex-stratified analyses to test the relationship between cholesterol catabolism and dementia.*

3. For Step 2, the authors might consider discussing the appropriateness of using matched LMT users as the control group for BAS users, in greater detail. In particular, what might be some considerations leading to a patient being prescribed LMT instead of BAS (or vice versa)? This is particularly since the matching was performed based on relatively few demographics (sex, year of birth, region, year of clinic registration/first prescription)

We have added additional details/rationale on the use of matched LMT users in the Methods section to better clarify the considerations when using this group as a control. We selected non-statin LMT users as an active comparator because, similar to BAS, non-statin LMTs are often used independently or in combination with statins.

Because patient comorbidity profiles can influence a clinician’s decision to prescribe BAS *vs.* LMTs, we compared the frequency of relevant clinical events in the 12 months prior to first BAS/LMT prescription in the two groups (supplemental Table 5). We then adjusted for factors that were different by the 2 groups to account for possible confounding-by-indication.

*BAS is often used as a second-line treatment independently or in combination with statins and therefore we selected non-statin LMT users as an active comparator group. In both groups (BAS or non-statin LMTs), we allowed for prior statin use in combination with either BAS or LMTs.*

*Models were adjusted for factors that were significantly different between BAS and LMT groups to account for potential confounding by indication. These factors included smoking status, body mass index (BMI), alcohol consumption, prior metformin use, coronary artery diseases, type 2 diabetes, dyslipidemia, and cancer history.*

4. For Step 2, a correlation was found between BAS use and VaD for males, but not between BAS use and any dementia/AD/other dementia, even for males. From line 120, the hypothesis inspired from Step 1 was whether BAS would alter risk of (various types of) dementia. As such, it might be discussed further as to whether there are any characteristics particular to VaD that might have given rise to this correlation, all the more due to the acknowledged limitation of likely inaccuracy in clinical diagnoses of dementia subtypes in primary care settings (line 481).

While the associations between BA and WML may partially explain associations with BAS use and VaD, we agree with the reviewer that there are likely inaccuracies in clinical diagnoses of dementia subtypes in large clinical datasets like CPRD. We have added a table (Supplementary Table 6) indicating any differences between individuals with VaD, AD, other dementia NOS, and any dementia. Patients who developed VaD were more likely to have previous coronary artery disease (11% *vs.* 6%, p=0.04) and more likely to have used metformin (14% *vs.* 9%, p=0.05) compared with individuals who developed AD.

We have also discussed the important role of ‘mixed brain pathologies’ in driving dementia outcomes. Several longitudinal studies have consistently demonstrated the coexistence of multiple pathologies and common occurrence of both AD and vascular pathology in older individuals with dementia.

*It is also important to note that large longitudinal studies have consistently reported that mixed brain pathologies account for the majority of dementia cases with considerable overlap between AD neuropathology and vascular brain injury including macroscopic, lacunar and microscopic infarcts [46, 47].*

5. For Step 3, given the major role played by sex-based stratification in the previous analyses, it might be appropriate for the relevant analyses and results (Figures 5 & 6, Supplementary Tables 7 & 8) for this step to be stratified by age too.

We have included age in all models in Step 3. We have additionally replaced bulk tissue array-based transcriptomic analyses with singe cell RNA sequencing (scRNA-Seq) data from AD and CN samples from ROSMAP. This larger sample size allowed us to sex-stratify our results; we have presented male and female results side-by-side in a heatmap to clearly indicate sex differences in brain gene expression of BA receptors in AD versus control.

For the results from the BLSA post-mortem study, because of sample size constraints driven by the small number of individuals with brain tissue BA metabolite data above the limit of detection (LOD), we were unable to perform sex stratified analyses. We have indicated this limitation in the Methods section and have also acknowledged this as a limitation in the Discussion section.

*Due to a small number of individuals with BA metabolite values above LOD, we were not able to sex-stratify these analyses.*

*Limitations of our study include the relatively small sample sizes in the BLSA-NI and autopsy samples. Additionally, we were unable to sex-stratify analyses of brain tissue BA concentration due to a limited number of individuals with BA metabolite concentration values above LOD.*

6. It is stated that "these findings are among the first to demonstrate sex-specific associations between the rate-limiting step in primary BA synthesis and brain amyloid deposition as well as longitudinal changes in brain WML burden" (line 411). Given the significance of the sex-specific analyses throughout, it might be appropriate to discuss any other work suggesting possible sex-specific associations/mechanisms. The existing discussion does not appear to propose any plausible explanation for the observed sex-specific associations through the underlying mechanism.

In addition to adding rationale for the *a priori* decision to explore sex-specific associations in the Introduction section, we have expanded the Discussion section to more comprehensively discuss plausible mechanisms/ pathways explaining the observed sex-specific associations.

*While we have not addressed the precise mechanisms underlying sex-specific associations between BA metabolism and dementia pathogenesis, prior evidence suggests important sex differences in lipid metabolism that impact risk of cardiovascular disease [42, 43]. These effects extend to differences in cholesterol catabolism and sex-specific responsiveness to therapeutic lowering of cholesterol levels by increasing its conversion to BAs. Animal studies have also shown sex specific differences in bile acid homeostasis during aging and suggest that these may be mediated by differences in expression of BA transporters as well as CYP7A1, the rate-limiting enzyme in BA synthesis [44].*

**Reviewer 2**

1)      Were the samples collected from fasting patients? The information seems to be missing in the text.

Patients were fasting at blood draw. We have added this detail to the Supplementary text associated with the Methods section.

*Quantitative metabolomics assays were performed on serum samples collected after 2009 among BLSA-NI participants after an overnight fast…*

2)      As acknowledged by the authors the smaller N in this cohort may be a limitation and sex-specific analyses can further reduce power. It would be therefore very relevant to have data from other cohorts, ie ADNI, where some BAs were previously measured but sex stratification analyses were not provided (PMID: 30337152).

We appreciate this suggestion from the reviewer. We have now included analyses of serum BA levels and neuroimaging outcomes from ADNI to validate our index findings in BLSA. Our validation analyses now include 1,666 ADNI participants with serum bile acid data and an average of 5.6 longitudinal MRI visits (8686 visits in total). Based on our index results from BLSA where we identified a sex-specific association between bile acids and neuroimaging outcomes, we explored this relationship in ADNI among these brain regions. We were thus able to confirm our bile acid specific findings in an independent cohort that was demographically different than BLSA. This validation provides further evidence for the sex specific association between bile acids and neuropathologic changes associated with dementia.

In the published paper (PMID: 30337152) authors did not explore longitudinal or sex stratified associations with brain atrophy; we agree with the reviewer that the addition of these results are extremely relevant.

3)      Were the analyses adjusted for use of medications for ie. cholesterol? This could also be very relevant and affect the analyses.

For BLSA, we have added statin use as a covariate in sensitivity analyses and described results in Supplementary Table 4. Results did not change substantially after the addition of the additional covariate.

4)      What are the levels of BAs in AD serum samples? Were the results from previous studies confirmed?

The BLSA-NI sample only includes cognitively unimpaired participants and therefore we are unable to examine relationships between levels of BAs and AD.

STEP 2
1)      Users of statins were excluded, I wonder if it could be relevant to investigate them as well in this study?

LMT or BAS users who also were statin users were included in this study as LMTs and BAS are often used as second-line treatments for hypercholesterolemia in combination with statins. We excluded statin only users as they were not on LMTs or BAS which were the medications of interest for this study. We have more clearly described this in the Methods section for Step 3.

*From the August 2018 CPRD data release, we identified patients ≥ 18 years old who had a first prescription record (i.e. new users) for BAS (colestipol, colesevelam, cholestyramine) or non-statin lipid modifying therapies (LMT; fibrate, cholesterol absorption inhibitor, nicotinic acid derivative and probucol) between January 1^st^, 1995 and August 1^st^, 2018. BAS is often used as a second-line treatment independently or in combination with statins and therefore we selected non-statin LMT users as an active comparator group. In both groups (BAS or non-statin LMTs), we allowed for prior statin use in combination with either BAS or LMTs. Individuals who only had a prescription record of statin use were excluded from this study.*

STEP 3

1)      LOD of BAs in measurement from autopsy brain may be a limitation. Would it be possible to measure amyloid beta levels as well? It would add important information.

While we do have CERAD and Braak scores for the autopsy brain samples, because of the limited number of samples with usable values above LOD (sample size range from n=5-9 depending on brain region), we were limited in going beyond assessing whether primary BAs were detectable in the brain or not. We have clearly indicated the limitations of the autopsy brain study in our expanded limitations section. For future studies we are very interested in increasing the sample size in the BLSA autopsy cohort in order to assess associations with pathology and other outcomes as suggested by the reviewer.

*Limitations of our study include the relatively small sample sizes in the BLSA-NI and autopsy samples. Additionally, we were unable to sex-stratify analyses of brain tissue BA concentration due to a limited number of individuals with BA metabolite concentration values above LOD.*

2)      Use of data from GEO datasets to prove hypothesis. The authors are strongly suggested to perform qPCR on autopsy brain from BLSA cohort to investigate and confirm the changes in gene expression observed in datasets from GEO. They should use for this the same regions used for BAs measurements (brain areas used for gene expression of FXR and TGR5 are not the same, nor the same cohort).

We are grateful for this excellent suggestion. We have significantly expanded our analyses of brain gene expression of BA receptors by:

i) Analyzing single cell RNA-Seq data from ROSMAP samples to examine cell type specific changes in BA receptor gene expression in AD versus Controls samples.
ii) Including all characterized BA receptors to derive a comprehensive understanding of changes in gene expression related to alterations in brain BA signaling in AD
iii) Analyzing sex-specific differences in brain BA receptor gene expression.

3)      Gene expression levels of receptors may not be enough to support hypothesis on mechanisms and at least protein levels should be also measured.

We thank the reviewer for this excellent suggestion. It would indeed be of great interest to match the gene expression profiles from scRNA-Seq with their corresponding protein level data. At present ROSMAP does not include single cell proteomic data. This is however a rapidly evolving technology and it is likely that we will eventually be able to acquire these data at the single cell resolution in future studies.

4)      The results from STEP 3 do not support sex differences observed in Step 1 and 2, which are not explored. The authors should run analyses in males and females and if possible, increase the N for this study.

While we were unable to examine sex differences in the BLSA post-mortem cohort because of sample size constraints, we have expanded our analyses of brain gene expression of BA receptors by analyzing single cell RNA-Seq data from AD and CON participants from the ROSMAP study. We have analyzed sex-specific differences in this significantly expanded study.

In general:
1)      The differences in male and females in STEP 1 and 2 are the main novel result, very interesting and should definitely be discussed more and investigated deeper. What could be the mechanisms behind? Is there literature that could support these sex-specific differences? What reported by the authors seems not exhaustive.

As indicated in our response to Reviewer 1 we have significantly expanded both the Introduction and Discussion to support our sex-specific analytic plan and more comprehensively discuss our sex-findings. Please see our response to Reviewer 1 Comment 2 and Comment 6.

**Reviewer 3**

(1) On the back of three experiments of varying design and mostly all low powered studies, the authors are making some fairly strong claims about dementia associations and mechanisms. However the data to support these claims are low powered (as they themselves recognize and point out within their limitations section), and the p values frequently borderline, and unlikely to survive correction for multiple testing (e.g., Table 1 showing associations of BA with brain volumetric measures; cDVR, precuneus DVR, WML, Table 2 testing association of BAS use and dementia risk; out of 24 tests, only one has a p value of < 0.05, which would not survive correction for multiple testing, Fig 5 testing brain BA concentration in control vs AD subjects; out of 6 tests only one has p<0.05). These data are at best suggestive, and the discussion should reflect this, rather than presenting strong and definitive claims about dementia associations and mechanisms. Having a limitations section is a
good idea, but does not obviate the need for a measured discussion, which makes claims in proportion to the strength of the data.

We thank the reviewer for this suggestion. In the revision we have significantly expanded our analyses to include the ADNI study (1,666 participants with serum BA data and an average of 5.6 longitudinal MRI visits (8686 visits in total)) and the ROSMAP study (46 participants with single cell RNA-Sequence (scRNA-Seq) data across eight brain cell types. We have corrected for multiple comparisons in all analyses using both datasets which provide us with converging evidence for a sex-specific association between BA metabolism and dementia.

In the following analyses, we controlled for multiple comparisons and only described results that were below the FDR-corrected threshold of p = 0.05: BLSA longitudinal associations between serum BA concentrations and rates of brain atrophy; ADNI longitudinal associations between serum BA concentrations and rates of brain atrophy; single-cell RNA sequence (scRNA-seq) sex-specific analyses of differences in BA receptor gene expression between AD and CON.

We have modified the Discussion section to more conservatively reflect the results based on the strength of the data and also have expanded the limitations section to more clearly address considerations of multiple hypotheses testing. acknowledge

While we acknowledge the limitations of not controlling for multiple comparisons particularly in analyses with low sample size, we believe that the addition of an independent validation sample (ADNI) as well as the use of a large scRNA-Seq dataset from ROSMAP provides important confirmation of our index results and their interpretation in a mechanistic context relevant to dementia pathogenesis.

(2)  In several parts of the manuscript the authors claim to have used a "novel study design". However the three study designs they outline are commonly used study types. The authors can claim perhaps to have used 3 studies which each differ in design and/or methodology but should avoid claiming/overstating the novelty of the design or methodology.

We agree that each component of the study has been used extensively in prior AD/ dementia studies. However, we believe our approach is different in the use of the three steps in combination as a strategy to identify plausible drug targets in dementia. We believe that the combination of using metabolomics and transcriptomics data to identify a candidate biochemical pathway and testing whether pharmacological manipulation of this pathway alters dementia outcomes in a real-world clinical dataset is a particular strength of the study. In deference to the reviewer, we have removed the term “novel” study design in the revision. Additionally, we have more appropriately described the study design in the Discussion section.

*Our study design represents an approach for identifying biological mechanisms of risk associated with dementia as well as to discover potential targets for disease-modifying treatments. First, the use of targeted metabolomics and transcriptomics within longitudinal observational studies in combination with established markers of disease progression (e.g., amyloid accumulation and brain atrophy) enables the identification of specific biochemical pathways that may present plausible drug targets. Second, the use of large, real-world clinical datasets with dementia outcomes enables testing drugs that may impact such targets.*

Furthermore, I am uncertain as to whether combining three independent studies adds statistical power, or risks conflating potentially unrelated results? Using replication cohorts would have seemed a stronger approach, rather than using multiple small studies of orthogonal design. However a statistician would be better placed to address this question.

As suggested by both Reviewer 2 and 3, we have added two additional studies to the revision. First, we have included the ADNI study (1,666 participants with serum BA data and an average of 5.6 longitudinal MRI visits (8686 visits in total)). as an independent cohort specifically to validate our neuroimaging findings in BLSA. We have also included scRNA-seq data, a non-array based, cell specific dataset (46 Religious Orders Study and the Rush Memory and Aging Project (ROSMAP) participants with data across 8 major brain cell types in the aged cortex including inhibitory neurons, excitatory neurons, astrocytes, oligodendrocytes, microglia, oligodendrocyte progenitor cells, endothelial cells, and pericytes) to comprehensively test sex-specific BA receptor gene expression differences in AD vs CON samples.

(3)  The authors interpret the lower BA values they observe in males as a possible "mediator of early pathological changes in AD" (pg 18). However they do not canvas other possible explanations, such as secondary effects, resultant from neuronal cell death, brain atrophy or neuropathologies present.

In the Discussion section, we have included text indicating alternative explanations for the cross-sectional associations we observed.

*This relationship appears to be driven primarily by males suggesting a novel sex-specific association between BA synthesis and brain amyloid accumulation. It is important to note however, that these cross-sectional analyses are not able to determine whether pathology, brain atrophy or other dementia associated endophenotypes may modify cholesterol catabolism.*

In the case of the post mortem tissue, ease of metabolite extraction from control vs AD tissue might be another contributing factor (were internal standards used for the assay?).

We have added a detailed description on the use of internal standards in the metabolomics assays in the Supplementary text section.

*Biocrates internal standards*

*A defined, consistent amount of internal standard was applied to each sample before and calibration standard early in the sample preparation. An external 7-point calibration was included in each measurement unit. After measurement, peaks were extracted using Waters MassLynx software or Sciex Analyst® software. The peak annotation for the samples depended on the relation of retention time of the analyte peak signal to the peak signal of the internal standard. The target relation between these two factors for the samples was specified by the relation of both factors observed in the calibrator. The mass tolerance of the quadrupole devices was around 0.1 m/z. The retention time tolerance was below 0.02 min. The calibration curve is constructed as a plot of X = ratio of the concentration of analyte to concentration of internal standard versus Y = ratio of area of analyte to the internal standard. For application, the ratio of analyte to internal standard area is determined for the samples and the equation for the regression line then allows determination of sample concentration. If the sample volume used deviated from the target volume of the assay, a factor was included in the calculation to take the volume difference into account.*

Furthermore the negative associations of metabolites and brain atrophy in females are not discussed in any detail, nor why the marked difference between males and females, nor reasons for the heterogeneous associations of brain pathology and specific metabolite levels.

Based on recommendations from Reviewer 1 and 2, we have conservatively decided to focus primarily on the sex-specific association betweenmetabolite levels and dementia in males. As indicated in our response to Reviewer 1, we have significantly expanded both the Introduction and Discussion to support our sex-specific analytic plan and more comprehensively discuss our sex-specific findings. Please see our response to Reviewer 1 Comment 2 and Comment 6.

The rational for stratification by sex is also not entirely clear, since, of the three metabolites assayed, only 7α-hydroxycholesterol had a significant p-value of 0.034 (without correction for multiple testing) for the "total" group
(Table 1). Similarly in Table 2/supplementary Table 4 there are no significant differences in the "overall" analysis, so the rationale for stratification should be more clearly outlined.

Please see our response above.

Subject numbers should be shown within all tables and figures, both in the main text and supplementary section, and include the totals and numbers following sex stratification.

We have added sample size to all tables and figures. This has been included in either the actual table/ figure or the table/ figure legend. 

Supplementary Table 2 shows that there are no significant associations between any of the three serum metabolite concentrations and PiB PET status, whereas Table 1 in the main text (pg 12) shows some significant associations between cDVR (amyloid-β deposition) and 7α-hydroxycholesterol in males. These apparently conflicting outcomes should be discussed, and some explanation offered.

Our results indicate that there are no significant differences between metabolite concentrations in Amyloid+ve versus Amyloid-ve individuals. However, among Amyloid+ve individuals (total sample and males only), there are significant associations between metabolite levels and brain amyloid burden (both globally and within the precuneus- an early site of amyloid deposition in AD). These are two separate analyses, one testing group comparisons and the other testing associations only in the PiB/ Amyloid +ve group. We have clarified these distinct analyses in the Methods section.

*To test for group differences between amyloid +ve and amyoid -ve individuals, we tested associations between serum concentrations of metabolites (i.e. 7α-OHC, CA and CDCA) and brain amyloid deposition, in total and sex-stratified linear regression models with metabolite concentrations as the dependent variable and the binary amyloid variable (i.e. amyloid +ve/amyloid -ve) as the main predictor. Covariates included mean-centered age and sex in the total model, and mean-centered age only in the sex-stratified model. We next tested the association between metabolite concentrations and mean cDVR i.e. global brain amyloid burden (BLSA) and precuneus DVR (BLSA) in amyloid +ve individuals only. We used similar linear regression models, replacing the binary amyloid variable with the continuous DVR variable as predictors.*

It is a little odd that the authors discuss their findings in the context of "early neuroimaging markers", "early neurodegeneration", "early pathology changes", etc, since the majority of their subjects are >65 years of age, and well into the range of late onset dementia.

In STEP 1 in BLSA as well as STEP 3 in CPRD we were explicitly interested in testing associations prior to the onset of clinical symptoms or disease diagnosis. We have clarified this by removing the use of the term “early” in the Discussion section and more appropriately framed our results.

*In summary, we have combined targeted metabolomic assays of serum with in vivo amyloid PET and MR imaging of the brain to identify cholesterol catabolism and BA synthesis as a biological pathway involved in neuropathological changes prior to dementia onset.*

The authors do not make a clear statement as to whether the changes observed here are likely specific to a particular dementia subtype (they mention BA associations in both AD and vascular dementia), a particular neuropathology (amyloid deposition, WMH) or may just be an age related phenomenon, increasing in parallel with increase of neuropathological features with age.

This is an important consideration also raised by Reviewer 1 Comment 4. The reviewer highlights a key observation emerging from multiple longitudinal studies indicating that “pure” dementia due to a single homogenous pathology is rare. The vast majority of dementias in older individuals are believed to be a manifestation of multiple, overlapping pathologies. We have included this important consideration in the manuscript and cite two key examples of observational studies that have established the role of multiple, co-occurring pathologies in dementia.

*It is also important to note that large longitudinal studies have consistently reported that mixed brain pathologies account for the majority of dementia cases with considerable overlap between AD neuropathology and vascular brain injury including macroscopic, lacunar and microscopic infarcts [46, 47].*

Table 2 and supplementary Table 4 appear to have the same data, but supplementary Table 4 has more detail. I suggest replacing Table 2 with supplementary Table 4 in the main manuscript.

We have replaced Table 2 with the expanded supplementary Table 2.

The Figure 3 flow chart shows that n=3,208 BAS users and  23,483 LMT users were available from the CPRD dataset. However were all these subjects used in the analyses presented here? It is a bit difficult to ascertain since subject numbers were not shown in most figures and tables (see previous comment - they should all be shown) - however the "N cases" in supplementary figure 4 have much smaller numbers. If fewer numbers were used for analysis than shown at the bottom of figure 3, then it would be useful to extend this flow chart to show the number of BAS and LMT users that were included in the analyses performed in the current study.

We thank the reviewer for this comment. The number of cases in the updated Table 2 represent numbers of patients in each subgroup with the outcome (i.e. any dementia, VaD, AD, and Other Dementia, NOS). To clarify we have now included the sample size with event/ total number; the total number sample size in the table now corresponds with the sample size indicated in the flow chart.

The authors are using a non-conservative approach to data/results interpretation, frequently claiming that specific associations were established, when only uncorrected data have p<0.05 but correction for multiple testing /FDR values not are statistically significant. Occasionally they even claim to observe changes when the uncorrected p-value is ≥0.05. This approach is particularly evident on pg 14 (results section). While they could speak of suggestive changes or data trends, however data which does not survive correction for multiple testing, or has p-values ≥0.05 should be reported as not significant. There is otherwise a risk of overinterpreting weak data.

Please see our response to Reviewer 3 Comment (1) above.

Figure 6 would more clearly represent the level of difference between control and AD bile acid gene expression if the y-axes were presented as non-transformed data, rather than log2 data.

We have replaced GEO data and Figure 6 with single-cell RNA-Seq data from ROSMAP and a heatmap indicating sex-specific differences between AD and CON.

The last paragraph on pg19 beginning "Our study design represents…." could be deleted,  since a detailed outline of the three studies was provided earlier in the manuscript.

We have edited this paragraph as below

*Our study design represents an approach for identifying biological mechanisms of risk associated with dementia as well as to discover potential targets for disease-modifying treatments. First, the use of targeted metabolomics and transcriptomics within longitudinal observational studies in combination with established markers of disease progression (e.g., amyloid accumulation and brain atrophy) enables the identification of specific biochemical pathways that may present plausible drug targets. Second, the use of large, real-world clinical datasets with dementia outcomes enables testing drugs that may impact such targets.*
