# Supplementary material for: Bile acid synthesis, modulation, and dementia: A metabolomic, transcriptomic, and pharmacoepidemiologic study
Source: PLoS Med. 2021 May 27;18(5):e1003615. doi: 10.1371/journal.pmed.1003615 (PMC8158920; doi:10.1371/journal.pmed.1003615)
Supplement: Attachment — Submitted filename: Response_to_reviewers_R2_12.12.2.docx [file pmed.1003615.s014.docx]

Note: reviewer comments are indicated in black text. Our response to reviewer recommendations are indicated in red text immediately below each comment. We have additionally included relevant text from the revised manuscript below each response in *red italics.*

Requests from the editors:

1- Please report your study according to the relevant guideline(s), which can be found here: <http://www.equator-network.org/> Please include the completed checklist as Supporting Information. When completing the checklist, please use section and paragraph numbers, rather than page numbers. Please add the following statement, or similar, to the Methods: "This study is reported as per the XXX guideline (S1 Checklist)." As this study uses multiple designs, I am leaving it to you to choose the appropriate guideline(s).

We have added the completed STROBE checklist as supporting information and have included the appropriate statement in the Methods section.

*This study is reported as per the STROBE guidelines (Supplementary Table 1).*

2- In your data statement, please include accession numbers or DOIs necessary for researchers to request the specific datasets used in this study.

We have included appropriate accession numbers/ DOIs for all publicly available datasets used in the study. We have also included a data availability statement on how to access BLSA, ADNI, and CPRD data.

***Data availability***

*Baltimore Longitudinal Study of Aging (BLSA) data are available to researchers and can be requested at* [*https://www.blsa.nih.gov/researchers*](https://www.blsa.nih.gov/researchers)*.*

*Rush Memory and Aging Project (ROSMAP) data can be requested at* [*https://www.radc.rush.edu*](https://www.radc.rush.edu)*; ROSMAP data used in this study is available at:* [*https://www.synapse.org/#!Synapse:syn18485175*](https://www.synapse.org/#!Synapse:syn18485175) *under the doi 10.7303/syn18485175*

*Clinical Practice Research Datalink (CPRD) data is available to researchers and can be requested at* [*https://www.cprd.com/public*](https://www.cprd.com/public)*.*

3- Thank you for editing the reference call-outs. Please also remove the space between reference numbers (e.g., [1,2]).

We have removed the space between reference numbers.

4- Thank you for editing the title, however, please edit further to fit PLOS Medicine’s style. Titles cannot be declarative. Additionally, while we realize that this is a unique multi-step study design, but a briefer description of the study design would be preferred.

We have updated the title to the following: Bile acid synthesis, modulation and dementia: a metabolomic, transcriptomic and pharmacoepidemiologic study

* Throughout the manuscript, please remove causal language (e.g., “increases risk of” at line 72 should be “was associated with”).

We have removed all causal language from the paper and replaced it with language appropriately indicating the association between predictors and outcomes.

5- Please edit the Abstract Background section to replace the summary of the 3 step study design with a brief statement of the main study question. The study design should be introduced in the Abstract Methods and Findings section.

We have moved the 3-step study design to the Methods and Findings section and have replaced it in the Background with the main study question.

***Methods and Findings***

*We used a 3-step study design to examine the role of the primary BAs, cholic acid (CA) and chenodeoxycholic acid (CDCA) as well as their principal biosynthetic precursor, 7α-hydroxycholesterol (7α-OHC), in dementia. In Step 1 we tested whether serum markers of cholesterol catabolism were associated with brain amyloid accumulation, white matter lesions (WMLs) and brain atrophy. In Step 2 we tested whether exposure to bile acid sequestrants (BAS) was associated with risk of dementia. In Step 3, we examined plausible mechanisms underlying these findings by testing whether brain levels of primary BAs and gene expression of their principal receptors are altered in AD.*

6- Abstract Methods and Findings:
* Please include the population and setting, years during which the study took place, length of follow up, and brief demographic details (e.g., age, sex) for each cohort included.
* Please quantify the main results (with 95% CIs and p values).
* Before describing the positive association at line 71, lease add a sentence describing the negative findings quoted around line 505.
* Please remove the last sentence (“Our findings merit confirmation…”) or move it to the Abstract Conclusions

To the Abstract Methods and Findings, we have added brief demographic details for all cohorts included in the study and have quantified results with p-values and H.R./ 95% CI were appropriate. We have also included the negative findings and have removed the last sentence.

*The BLSA is an ongoing, community-based cohort study that began in 1958. Participants in the BLSA neuroimaging sample were approximately 46% male with a mean age of 76 years; longitudinal analyses included an average of 2.5 follow-up MRI visits…*

*We found that BAS use was not significantly associated with risk of all-cause dementia (HR=1.03, 95% CI=0.72-1.46, p=0.88) or its subtypes. We found a significant difference between the risk of vascular dementia (VaD) in males compared to females (p=0.040) and a significant dose-response relationship between BAS use and risk of VaD (p-trend=0.045) in males.*

7- The Abstract Conclusions are still a bit strong, given the limitations of this study. Please address the study implications without overreaching what can be concluded from the data; the phrase "In this study, we observed ..." may be useful.
* Please interpret the study based on the results presented in the abstract, emphasizing what is new without overstating your conclusions.
* Please remove the last sentence “Targeting brain primary BA signaling...”

We have made all recommended edits to the Abstract Conclusions and have made sure not to overreach with the study conclusions.

*We observed that lower serum BA concentration mainly in males is associated with neuroimaging markers of dementia and pharmacological lowering of BA levels may be associated with higher risk of vascular dementia in males. We hypothesize that dysregulation of BA signaling pathways in the brain may represent a plausible biologic mechanism underlying these results. Together, our observations suggest a novel mechanism relating abnormalities in cholesterol catabolism to risk of dementia.*

8- Figure 2 can be removed, and Figure 1 can be a supplementary figure.

We have moved Figure 1 to the supplementary section and have removed Figure 2.

*The principal catabolic fate of cholesterol is its conversion to the primary bile acids (BAs), cholic acid and chenodeoxycholic acid (CA and CDCA respectively) through BBB-permeable intermediate metabolites called oxysterols (Supplementary Figure 1).*

9- In the Methods section, please include a section on ethics approval, specify the ethics boards/committees that provided approval for each of the cohorts and studies included (rather than “The local Institutional Review Board...”, and specify how consent was obtained (or who waived consent, if that was the case).

We have created a separate section with a detailed summary of ethics approval and how consent was obtained for all 4 cohorts/ populations used in the study: BLSA, ADNI, ROSMAP, CPRD.

*Ethics approval*

*The BLSA study protocol has ongoing approval from the Institutional Review Board (IRB) of the National Institute of Environmental Health Science, National Institutes of Health (“Early Markers of Alzheimer’s Disease (BLSA)”, IRB No. 2009-074). Informed written consent was obtained at each visit from all participants.*

*The ADNI study protocol was approved by the IRBs of all the participating institutions/study sites [5]. Informed, written consent was obtained from all participants at each site. All ADNI studies are conducted according to Good Clinical Practice guidelines, the Declaration of Helsinki, and U.S. 21 CFR Part 50 (Protection of Human Subjects), and Part 56 (Institutional Review Boards). Additional details can be found at* [*adni.loni.usc.edu*](http://adni.loni.usc.edu)*.*

*The ROSMAP study, including the parent study and sub-studies, were approved by the IRB of Rush University Medical Center. Informed, written consent was obtained for all participants as well as an Anatomical Gift Act, and a repository consent to share data and biospecimens.*

*CPRD data is anonymized and general practitioners do not need to seek patient consent when sharing data with CPRD, and patients have the option of opting out. Additional details can be found at* [*https://www.cprd.com/public*](https://www.cprd.com/public)*. This study was approved by the CPRD Independent Scientific Advisory Committee (ISAC; Protocol # Protocol 18_173) and exempted from full institutional review board review by the National Institutes of Health Office of Human Subject Research.*

10- Line 653, “(fig-xx)” is this a reference to a figure to be added?

This was in reference to Figure 4. We have edited this error.

*… that we find to be differentially expressed in AD brains in a sex-specific manner (Figure 4).*

11- Final sentence, lines 712-714, please be a bit more specific on the implications for clinical practice and drug discovery.

We have edited the final sentence to focus more specifically on future experimental studies that may plausible therapeutic targets.

*Our findings suggest that future experimental studies may provide insight into modulation of BA levels as a plausible therapeutic target in dementia.*

12- Please move the funding information from the main text to the Financial Disclosure section of the metadata.

We have moved funding information to the financial Disclosure section.

13- At the end of the main text, please provide a list of all supplementary documents with titles and legends where applicable. Please also make sure all supplementary documents are referenced when relevant to the main text.

We have added a list of supplementary documents (text, tables, and figures) including titles and legends to the end of the main text and have made sure all have been referenced in the main text.

Comments from the reviewers:

Reviewer #1: We thank the authors for largely addressing our concerns from the previous review round. A fairly major concern however remains with the sex-specific associations claimed with CA and CDCA. In particular, with reference to the additional confirmatory ADNI analysis as presented in Table 1b, the impression gained at first glance is that the associations with CA and CDCA are universal (from all variables being significant in the "Total (n=1666)" column). Indeed, looking at the pvals, it appears likely that the non-significance of many variables once broken down into male/female subsets, might have arisen largely due to the reduced sample size.

Furthermore, while there appear to be far more CA/CDCA variables showing significance in Table 1b for males as compared to females, it is notable that all these nine variables have pval (FDR)=0.049, which appears to be just marginally below the significance threshold of p=0.05. In other words, were the significance threshold changed by merely 0.002, the conclusion reached would have been that neither CA nor CDCA variables had been shown to have had any associations with the rate of brain atrophy for males. Given that p=0.05 is a somewhat arbitrary, if traditional, measure (see for example "The reign of the p-value is over: what alternative analyses could we employ to fill the power vacuum?", L.G. Halsey, Biology Letters, 2019), it might remain slightly problematic to claim evidence of general CA/CDCA sex-specific associations as a major finding on this basis. Nonetheless, we believe the rest of the analysis to have merit.

We thank the reviewer for this important point. With regard to the ADNI findings based on the associations in the total and sex-specific findings, we agree that considering the findings in the total sample, likely some of the lack of associations in the male and female specific findings may be driven by reduced sample size. We have added this important point to the revised Discussion.

Considering the male and female effects separately, and the 0.049 false discovery rate (FDR): the false discovery date rate is generated by first sorting raw p-values and then calculating the minimum value alpha such that pk<alpha*m/k (where pk is the kth p-value in the sorted list and m is the total number of comparisons). Therefore, it is possible that multiple raw p-values (ours range from 0.005-0.018) may have an identical FDR threshold. This is important to note because while the FDR threshold is only modestly below 0.05 for several of the comparisons referred to in Table 1b by the reviewer, the raw p-values for those comparisons are generally far lower in the male-only samples than the female-only samples. This is therefore suggestive of a sex-specific association between BAs and neuroimaging outcomes in males. Furthermore, our observations of sex-specific effects are not restricted to neuroimaging outcomes but are also consistent with the results of our pharmacoepidemiologic analyses as well as those from brain transcriptomics.

We do however agree that our conclusions considering the ADNI neuroimaging analyses need to consider that the male-specific effect is modest and that the lack of sex-specific associations compared to associations in the total sample may be driven partially by sample size. We have edited the Abstract to more appropriately indicate these results and have also edited the Discussion accordingly.

*In ADNI, we found a modest sex- specific effect indicating that lower serum concentrations of CA and CDCA were associated with faster brain atrophy (FDR p=0.049) in males.*

*These findings were partially confirmed in ADNI where lower BA concentrations were associated with faster brain atrophy rates across several brain regions in males with far fewer associations in females. It is important to note however that the lack of sex-specific associations compared to the total sample in ADNI may be partially driven by sample size.*

Minor issue: in Line 179, "Religions Orders" might be "Religious Orders".

We have edited this error.
