# Supplementary material for: Bile acid synthesis, modulation, and dementia: A metabolomic, transcriptomic, and pharmacoepidemiologic study
Source: PLoS Med. 2021 May 27;18(5):e1003615. doi: 10.1371/journal.pmed.1003615 (PMC8158920; doi:10.1371/journal.pmed.1003615)
Supplement: Attachment — Submitted filename: Response_to_reviewers_R3_3.1.21.docx [file pmed.1003615.s015.docx]

Note: reviewer comments are indicated in black text. Our response to reviewer recommendations are indicated in red text immediately below each comment. We have additionally included relevant text from the revised manuscript below each response in *red italics.*

**Requests from Editors**:

In your data statement, please use "data are" consistently. Are all sources of data for your study quoted here (ADNI appears to be absent)? It appears that information on ADNI needs to be moved from the Acknowledgements section at the end of the paper.

We have ensured that data is used as a plural subject/ object consistently in the paper. We have moved ADNI data availability information from Acknowledgements to the Data Availability section and have also transferred that information to the appropriate location in the submission form (based on reviewer recommendations below).

At line 91, should "CN" be "CON"?

We have edited CN to CON in all instances.

At line 133, please remove "Finally ..."; and we suggest adapting the wording to "... levels of gene expression of BA receptors were altered ...".

We have made the recommended edits.

*We found that primary BAs were detectable in the brain and levels of gene expression of BA receptors were altered in AD mainly in males.*

In your Introduction around lines 160-175, please integrate "STEP 1" and so on into the text in a single paragraph; e.g., "First, we used targeted metabolomics assays ... Finally, we explored plausible molecular mechanisms ...".

We have made the recommended edits.

At line 539, we ask you to adapt "marginally increased risk" to "non-significantly elevated risk" (removing the subsequent "(non-significant)", noting the p value of 0.06.

We have made the recommended edits.

*BAS use was associated with non-significantly elevated risk of VaD in males (HR=2.89, 95% CI=0.96-8.68, p=0.06).*

In the paragraph summarizing the study findings at the start of your Discussion section, please make that "were/was associated" at lines 601/603.

We have made the recommended edits

*We found that lower serum concentrations of the rate-limiting biosynthetic precursor of BA synthesis, i.e. 7α-OHC as well as the primary BAs mainly in males were associated with neuroimaging measures of dementia progression and that pharmacological lowering of BA levels was associated with higher risk of vascular dementia in males* 

Please remove the information on data access from the end of the main text. This information will appear in the article metadata, via entries in the submission form.

We have removed the data availability/ data access section from the end of the main text and included the updated information on all studies included in the paper in the entry in the submission form.

In the reference list, please ensure that journal names are abbreviated consistently (e.g., "J Alzheimers Dis." for reference 2 and others). 

We have updated these references.

For reference 22 and any other preprints cited, please substitute "[preprint]" for the sentence about the absence of peer review.

Reference 22 has now been published. We included the update reference.

For reference 44, please make the journal name "PLoS ONE".

We have updated this reference.

Please break the STROBE checklist out into a separate attached file, labelled "S1_STROBE_Checklist" or similar and referred to by this label in your Methods section.

We have made this recommended edit.

*This study is reported as per the STROBE guidelines (see supplementary file: S1_STROBE_Checklist).*

**Comments from Academic Editor:**

The authors should improve their discussion of what the implications of sex-specific pathways might be, the magnitude of possible differences, and how this might fit with our understanding of descriptive epidemiological estimates of dementia.

We have expanded our discussion of sex differences and potential implication on epidemiologic estimates of dementia.

*It is important to consider these findings together with animal studies that have also shown sex specific differences in bile acid homeostasis during aging and suggest that these may be mediated by differences in expression of BA transporters as well as CYP7A1, the rate-limiting enzyme in BA synthesis [44]. It is likely that such differences are relevant in other biological pathways as well. Our own prior work has uncovered striking sex differences in the systemic inflammatory response in preclinical AD that is related to neurodegeneration [45] as well as differences in glucose metabolism that are associated with AD pathology [46]. These findings may have implications for testing targeted treatment interventions that take into consideration sex-specific differences in molecular mechanisms underlying AD pathogenesis. Understanding sex differences in biological pathways related to AD risk and progression may also have important implications in our understanding of descriptive epidemiological estimates of dementia that reveal sex-specific longitudinal differences in both prevalence and incidence of dementia in diverse cohorts [47]. It is also worth noting in this context that sex as a biological variable (SABV) has been largely ignored in neuroscience and dementia research [48,49].*

The authors might also want to consider making stronger statements about the fact that so much research doesn't report or address sex

We have indicated the limited research in AD/ dementia regarding sex differences. Please see above for relevant text.

I'd like the authors to acknowledge literature that suggests their ideas of circumscribed AD, VaD are out of kilter with what's observed in usual dementia in the oldest age groups, thus contributing to the perpetuation of identifiable clean subtypes (reasonable for younger old, not for older old).

We have expanded the Discussion to include additional text on mixed pathology in the oldest old and appropriate references.

*Additionally, particularly in the oldest old, “single neuropathological entities” [53] may be less relevant compared to mixed pathologies including AD and vascular disease [54-56].*

**Comments from Reviewers:

*** Reviewer #1:** 

We thank the authors for clarifying our main concern about the FDR pvals, and reflecting a slightly more nuanced treatment in the Abstract and Discussion.

The claim however in the response that "the raw p-values for those comparisons are generally far lower in the male-only samples than the female-only samples" might however then be supported by these raw p-values, perhaps in the Supplementary material. Moreover, it might be explained as to why these raw p-values were not initially used for analysis in this case since they supposedly demonstrate sex-specific differences that much more clearly, and why the minimum alpha method of calculating false discovery rate was used instead.

We have included the raw p-values in addition to the FDR-corrected p-values in Table 1a and 1b. We decided to indicate FDR-corrected p-values particularly in our brain volume (neuroimaging) analyses in both BLSA and ADNI to account for the possibility of Type I error due to multiple comparisons.
